# Glucose 6‐phosphate dehydrogenase variants increase NADPH pools for yeast isoprenoid production

**DOI:** 10.1002/2211-5463.13755

**Published:** 2024-01-02

**Authors:** Sri Harsha Adusumilli, Anuthariq Alikkam Veetil, Chinmayee Choudhury, Banani Chattopadhyaya, Diptimayee Behera, Anand Kumar Bachhawat

**Affiliations:** ^1^ Department of Biological Sciences Indian Institute of Science Education and Research Mohali Manauli India; ^2^ Department of Experimental Medicine and Biotechnology PGIMER Chandigarh India; ^3^ Department of Earth and Environmental Sciences Indian Institute of Science Education and Research Mohali Manauli India; ^4^ Present address: Department of Chemical and Biological Engineering University of Wisconsin‐Madison WI USA; ^5^ Present address: Department of Chemistry and Biomedical Sciences Linnaeus university Kalmar Sweden

**Keywords:** glucose 6‐phosphate dehydrogenase, isoprenoids, NADPH, *Saccharomyces cerevisiae*, sclareol, ZWF1

## Abstract

Isoprenoid biosynthesis has a significant requirement for the co‐factor NADPH. Thus, increasing NADPH levels for enhancing isoprenoid yields in synthetic biology is critical. Previous efforts have focused on diverting flux into the pentose phosphate pathway or overproducing enzymes that generate NADPH. In this study, we instead focused on increasing the efficiency of enzymes that generate NADPH. We first established a robust genetic screen that allowed us to screen improved variants. The pentose phosphate pathway enzyme, glucose 6‐phosphate dehydrogenase (G6PD), was chosen for further improvement. Different gene fusions of G6PD with the downstream enzyme in the pentose phosphate pathway, 6‐phosphogluconolactonase (6PGL), were created. The linker‐less G6PD‐6PGL fusion displayed the highest activity, and although it had slightly lower activity than the WT enzyme, the affinity for G6P was higher and showed higher yields of the diterpenoid sclareol *in vivo*. A second gene fusion approach was to fuse G6PD to truncated HMG‐CoA reductase, the rate‐limiting step and also the major NADPH consumer in the pathway. Both domains were functional, and the fusion also yielded higher sclareol levels. We simultaneously carried out a rational mutagenesis approach with G6PD, which led to the identification of two mutants of G6PD, N403D and S238QI239F, that showed 15–25% higher activity *in vitro*. The diterpene sclareol yields were also increased in the strains overexpressing these mutants relative to WT G6PD, and these will be very beneficial in synthetic biology applications.

## Graphical abstract

This study focuses on enhancing isoprenoid yields by increasing NADPH levels. Gene fusions of *S. cerevisiae* glucose 6‐phosphate dehydrogenase (*Sc*G6PD) with 6‐phosphogluconolactonase (6PGL) and truncated HMG‐CoA reductase were created, and overexpressing them yielded higher sclareol (diterpenoid). Rational mutagenesis of *Sc*G6PD identified mutants (N403D and S238QI239F) with 15–25% higher activity and overexpression of the N403D mutant also showed increased sclareol yields.

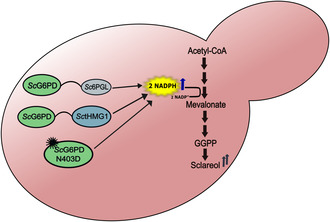

**Abbreviations**
6PGD: 6 phosphogluconate dehydrogenase
6PGL: 6 phosphogluconolactonase
G6P: glucose 6‐phosphate
G6PD: glucose 6‐phosphate dehydrogenase
GSH: glutathione
NADP: β‐nicotinamide adenine dinucleotide phosphate sodium salt hydrate
NADPH: nicotinamide adenine dinucleotide phosphate
WT: wildtype

Isoprenoids (terpenoids) are naturally occurring, biologically significant, diverse hydrocarbons derived from five‐carbon isoprene units. Many of these isoprenoids display important and valuable properties that result in their diverse applications. However, extraction and purification of these isoprenoids from their natural sources are neither economical nor sustainable. For this reason, intense efforts are being made to reconstitute these pathways in microbial hosts to enable their production in these organisms. *Escherichia coli* and *Saccharomyces cerevisiae* have been the hosts of choice for these plant‐derived isoprenoids.

Various efforts have been made to increase the specific isoprenoid yields in these organisms. The predominant approach has been to increase precursor molecule supply and direct the carbon flux into desired pathways. This has been combined with preventing the production of undesired metabolites from branched pathways [1].

Another important factor affecting yields is the limited supply of co‐factor NADPH (Nicotinamide adenine dinucleotide phosphate), a key co‐factor in the isoprenoid pathway. Increasing the NADPH pools in cells increased the yields of various products in diverse pathways, including isoprenoids. This has been demonstrated in the case of α‐santalene [2], sterols [3], xylitol [4], caffeine, and carotenoids [5] in yeasts reconstituted with these pathways. Some of the strategies for increasing NADPH levels have included the overexpression of some of the enzymes known to generate NADPH, such as glucose 6‐phosphate dehydrogenase (G6PD) [5], the overexpression of the regulators of the pentose phosphate pathway (STB5) [6], or, in the case of oleaginous yeasts, the overexpression of mannitol dehydrogenase (MDH2) [7]. Other approaches include increasing the flux into the pentose phosphate pathway [1] or the use of the NADH‐dependent HMG‐CoA reductase [8] (instead of the NADPH‐requiring enzyme of yeasts), which is the rate‐limiting step in isoprenoid biosynthesis in yeasts. However, increasing the efficiency of the enzymes catalyzing NADPH generation, which would impose a lesser burden than overexpression, is an approach that has surprisingly not been explored. Using enzymes with enhanced NADPH generation could be very beneficial for improving *S. cerevisiae* as a cell factory for isoprenoid production.

In this study, we have attempted to increase NADPH levels by improving the efficiency of the NADPH‐generating enzymes. Using a genetic screen that we developed, we evaluated different metabolic enzymes of *Saccharomyces cerevisiae* known to enhance NADPH levels. We found that glucose‐6‐phosphate dehydrogenase (G6PD), the first enzyme of the pentose phosphate pathway, was one of the best enzymes to target for further enhancement. Focusing on this enzyme, we evaluated multiple approaches. Firstly, we evaluated the orthologue of this enzyme in the red yeast *Rhodosporidium toruloides* known to make high levels of the isoprenoid carotenoid [9]. We also examined synthetic metabolon approaches (both nature mimics and those not found in nature) and a rational mutagenesis approach targeting residues in the active site pocket. The latter two approaches yielded promising variants, as seen through *in vitro* activity determinations as well as *in vivo* evaluations that included comparing the production of the heterogeneously produced diterpenoid sclareol in both the WT and the variants.

## Materials and methods

### Chemicals and reagents

All the chemicals were purchased from commercial sources and were either analytical grade or molecular grade. The growth media components were obtained from BD Difco (Franklin Lakes, NJ, USA) and Himedia (Mumbai, India). The amino acids, glutathione (GSH) (reduced), β‐nicotinamide adenine dinucleotide phosphate sodium salt hydrate (NADP), glucose 6‐phosphate (G6P), mevalonolactone and 6‐phosphogluconic dehydrogenase from yeast were obtained from Merck (Darmstadt, Germany). Zymolase‐20T was obtained from MP biomedicals (USA). Ultra centrifugal filters were obtained from Merck (Burlington, MA, USA). Oligonucleotides were obtained from Integrated DNA Technologies (IDT) and Merck (Bangalore, India). Vent DNA polymerase and restriction enzymes were obtained from New England Biolabs (Ipswich, MA, USA). Plasmid miniprep and gel/PCR clean‐up kits were purchased from Thermo Fisher Scientific (Waltham, MA, USA). The NADPH kit was obtained from Promega (Madison, WI, USA), nickel‐nitrilotriacetic acid agarose (Ni‐NTA), and polypropylene columns were obtained from Qiagen (Hilden, Germany).

### Strains, media, and growth conditions

The yeast strains used in the study are described in Table S1. The strains were maintained on yeast extract, peptone, and dextrose (YPD) medium and grown at 30 °C. The yeast cells were transformed by the Lithium acetate transformation method as described [10]; transformants were selected and maintained on synthetic defined (SD) minimal medium containing 0.17% yeast nitrogen base, 0.5% ammonium sulfate, and 2% glucose supplemented with leucine, lysine, uracil, and methionine at 80 mg·L^−1^.

The *E. coli* strain DH5α was used as a cloning host, and BL21(DE3)pLysS strain as a protein expression host were grown at 37 °C. The growth and handling of yeast and bacteria and all the molecular biology techniques used in this study were according to standard protocols [11].

### Cloning of genes into plasmid expression vectors

**Fig. 1:**
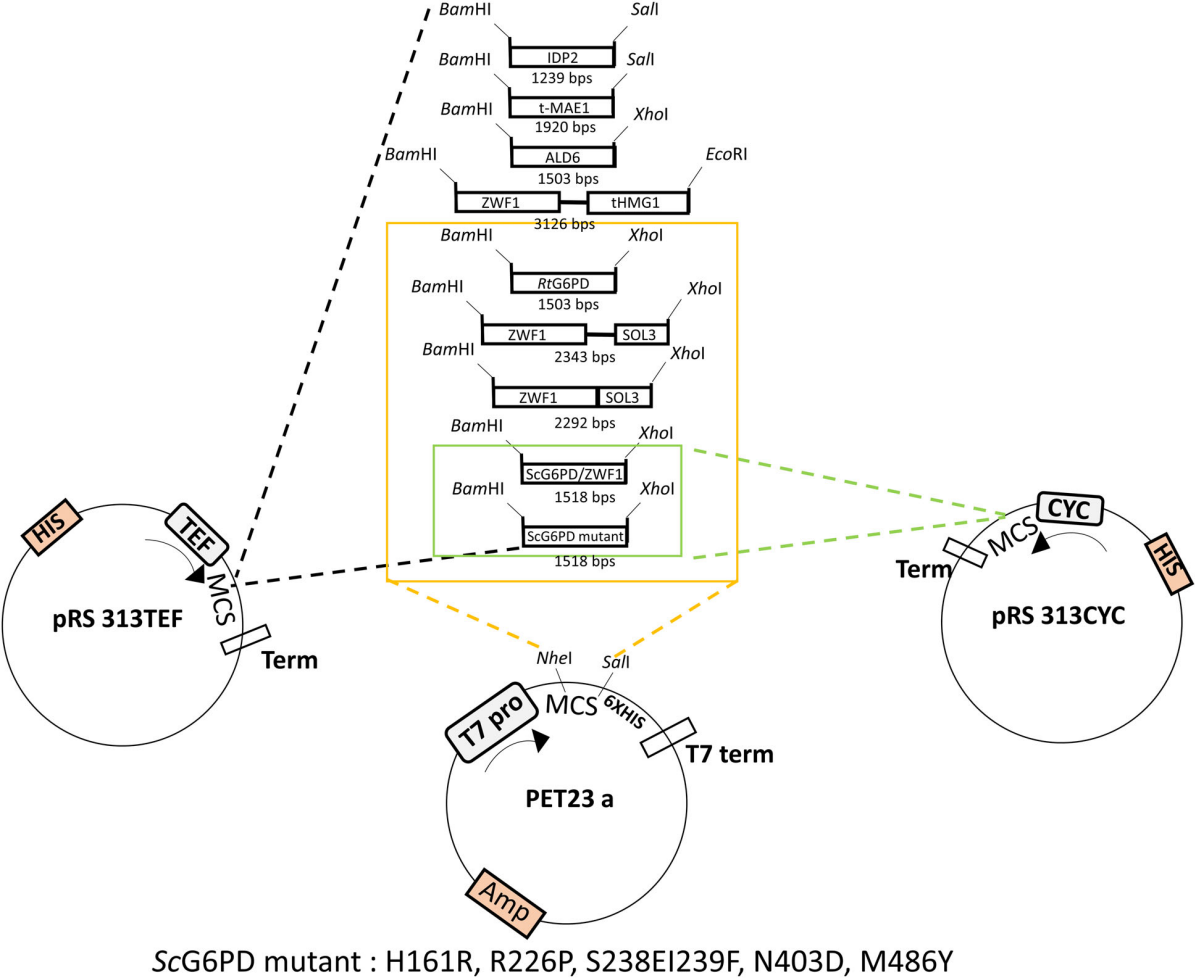
Schematic representation of cloning and construction of all the constructs mentioned in the study. Schematic representation of cloning and construction of all the constructs mentioned in the study. The construction of the constructs and cloning them into vectors pRS313TEF, pRS313CYC, PET23a were described in the text.

The genes *ZWF1* (which encodes G6PD), *IDP2*, *MAE1* with the first 90 bps truncated (t*MAE1*), and *ALD6* were amplified from genomic DNA isolated from the *S. cerevisiae* BY4741 strain with their respective forward and reverse primers, as shown in Table S2. The genes were cloned under the TEF promoter in a yeast centromeric vector pRS313TEF (the TEF2 promoter of *S. cerevisiae* is referred to as the TEF promoter, [12]) in the sites mentioned in Table S3. *ZWF1* and the *ZWF1* mutants were also cloned into the pRS313CYC vector, where the genes were under the weaker CYC promoter (the CYC1 promoter of *S. cerevisiae* is referred to as the CYC promoter, [12]). The vector, pRS313CYC, was created by excising the TEF promoter from pRS313TEF by *Xba*I and *Sac*I and replacing it with the CYC promoter. The gene *RtG6PD* was codon optimized and custom synthesized from GenScript (Piscataway, NJ, USA) (Accession no. OQ291226) and cloned into the yeast expression vector pRS313TEF. The G6PD‐6PGL and 6PGL‐G6PD fusion proteins were constructed by linking the *ZWF1* (5′ end) with the *SOL3* (3′ end), and the *SOL3* (5′ end) with the *ZWF1* (3′ end), respectively, by a nucleotide encoding a 16 amino acid poly Gly‐Ser linker by splice overlap extension‐polymerase chain reaction (SOE‐PCR). This SOE PCR consisted of three PCRs. The first PCR amplified the *ZWF1* gene with the primers *Sc*G6PD BamHI‐FP and G6PD‐link‐6PGL RP. The second PCR amplified the 6PGL gene using the primers G6PD‐link‐6PGL FP and 6PGL SalI‐RP. The third PCR was the joining PCR, which amplified the fusion protein from the first two PCR reaction products using the primers *Sc*G6PD BamHI‐FP and 6PGL SalI‐RP. The 2.3 kb fusion protein, along with the linker, was cloned into pRS313TEF and pET23 vectors (Novagen, Madison, WI, USA). The fusion protein G6PD‐6PGL without linker was also constructed by fusing *ZWF1* at the N‐term, immediately fused to *SOL3* at the C‐term, in the same way as mentioned above using *Sc*G6PD BamHI‐FP and G6PD‐6PGL RP in PCR1, G6PD‐6PGL FP and 6PGL SalI‐RP in PCR2. The G6PD‐6PGL was constructed using the products of PCR1 and PCR2 by *Sc*G6PD BamHI‐FP and 6PGL SalI‐RP and cloned into centromeric yeast expression vector pRS313TEF and bacterial expression vector pET23a (Novagen). The G6PD‐tHMG1 fusion protein was constructed by fusing G6PD at the N‐terminal with tHMG1 at the C‐terminal with an 11‐aa Gly‐Ser linker by SOE PCR. ZWF1 was amplified using *Sc*G6PD BamHI‐FP and *Sc*G6PD‐link‐*Sc*tHMG link RP; tHMG1 was amplified using *Sc*G6PD‐link‐*Sc*tHMG FP and *Sc*tHMG1‐ XhoI RP. Both of these amplicons were used to construct the G6PD‐tHMG1 fusion protein and cloned into pRS313TEF between the *Bam*HI and *Xho*I sites. The various mutants of *ZWF1* were constructed by splice overlap extension PCR (PCR1 with *Sc*G6PD NheI‐FP and corresponding mutant RP; PCR2 with corresponding mutant FP and *Sc*G6PD XhoI‐RP; the third PCR used PCR1 and PCR2 products as templates with *Sc*G6PD NheI‐FP and *Sc*G6PD XhoI‐RP primers) and cloned into pRS313TEF and PET23a vectors. The sclareol biosynthetic genes copal‐8‐ol diphosphate synthase (*CcCLS*) and sclareol synthase (*SsSS*) that were custom synthesized [13] were sub cloned into yeast centromeric vectors pRS314TEF and p416TEF, respectively, to make them compatible for a four‐plasmid yeast transformation system. All the clones constructed were confirmed by sequencing. The construction and cloning of the constructs used in the study are shown in Fig. 1. The plasmids used in the study are listed and described in Table S3.

The accession numbers of the primary nucleotide sequences used for the construction of plasmids used in this study are ZWF1: NM_001183079.1, *SOL3*: NM_001179294.1, *IDP2*: NM_001182061.1, *MAE1*: NM_001179595.1 and *ALD6*: NM_001183875.1. A truncated *HMG1* gene (t*HMG1*) lacking the membrane‐binding region (1–552 aa) was constructed from *HMG1*: NM_001182434.1.

### Dilution spotting of yeast cells

Yeast transformants were grown overnight in 5 mL of synthetic defined (SD) medium with the amino acids leucine, lysine, uracil, and methionine and then re‐inoculated into 10 mL of fresh media, grown to an OD_600nm_ of 0.8–1.0. The cells were harvested, washed with autoclaved water, and resuspended in sterile water at an OD_600nm_ of 0.2. These suspensions were serially diluted to 1 : 10, 1 : 100, and 1 : 1000. 10 μL of each suspension were spotted on the desired minimal medium plates containing different concentrations of methionine or reduced glutathione (Merck, Darmstadt, Germany, Cat No. G6529) and amino acid supplements. The plates were incubated at 30 °C, and the images were captured by the Bio‐Rad Gel Doc™ XR+ imaging system after 2–3 days.

### Expression and purification of proteins

The G6PD fusion enzymes and the G6PD mutants constructed were tagged at the C terminus with a 6X HIS tag and cloned into the PET23a expression vector. The expression vectors were transformed into *E. coli* BL21(DE3)pLysS. The transformants were grown in LB broth with 25 μg·mL^−1^ chloramphenicol and 100 μg·mL^−1^ ampicillin overnight and re‐inoculated into fresh culture at OD_600nm_ 0.05. The cultures were grown to an OD_600nm_ of 0.5, then induced with 0.5 mm Isopropyl β‐d‐1‐thiogalactopyranoside (IPTG) (Cat No. I2481C, Goldbio, St Louis, MO, USA) and incubated at 30 °C shaking for 5 h. The cultures were harvested, and the pellet was stored at −80 °C for further analysis.

The pellet was resuspended in lysis buffer [20 mm Tris–HCl buffer, pH 8 containing 10% glycerol, 500 mm NaCl, 1 mm phenylmethylsulfonyl fluoride, and protease inhibitor mixture (Cat No. P2714, Merck, Darmstadt, Germany)]. The cells were lysed by sonication at 20 amplitude, and 10 s sonication cycles were alternated with 15 s recovery periods. The sonicate was centrifuged at 10 000 ***g*** for 30 min at 4 °C, and the supernatant was collected. The cleared lysate was loaded onto a nickel‐nitrilotriacetic acid agarose column equilibrated with purification buffer (20 mm Tris–HCl, pH 8, 500 mm NaCl), and the supernatant was loaded onto the column. The bound protein was washed with purification buffer containing 30 mm imidazole and finally eluted in purification buffer containing 300 mm imidazole. The purified proteins were analyzed by SDS/PAGE (12% gel). The Microcon‐30 kDa Centrifugal Filter Unit (Merck, Burlington, MA, USA) was used to remove imidazole by buffer exchange (with purification buffer) and concentrate the protein. The concentrations of the purified proteins were estimated by Nanodrop (Eppendorf Bio Spectrometer^®^ Basic, Hamburg, Germany) and subsequently used in the enzyme assays.

### Determination of *in vitro* glucose 6‐phosphate dehydrogenase (G6PD) activities and kinetic parameters

The purified proteins were added to the assay reaction buffer (100 mm Tris–HCl, pH 8.0) containing 0.2 mm β‐Nicotinamide adenine dinucleotide phosphate sodium salt hydrate (NADP) (Cat No. N0505, Merck, Darmstadt, Germany), 0.01 m MgCl_2_, 0.6 mm glucose 6‐phosphate (G6P) (G7879, Merck, Darmstadt, Germany), and the reduction of NADP is monitored over time at 340 nm at 25 °C in the POLARstar Omega plate reader. The specific activities of the enzymes were calculated from the initial velocity, and 1 unit is defined as the enzyme required to reduce 1 μmol of NADP per minute at 25 °C. The enzyme activities at different pHs were carried out at pHs 5, 6, 7, 8, and 9 in different buffers. pH 5 and pH 6 were made with potassium phosphate buffer (100 mm), while pH 7, 8, and 9 were made with Tris buffer (100 mm).

The kinetic parameters were determined by varying the concentrations of G6P or NADP ranging from 5 to 300 μm, keeping the other substrate constant. The initial velocities obtained for each concentration were fitted to the Michaelis–Menten equation via non‐linear regression calculations, and the *K*_m_ values were obtained using graphpad prism 5.0 (Dotmatics, Boston, MA, USA).

### Detection of 6‐Phosphoglucono lactonase (6PGL) domain functionality *in vitro*

In this assay, the substrate of 6PGL, 6‐phosphogluconolactone, was determined by the action of the G6PD domain of G6PD‐6PGL on the substrate glucose 6‐phosphate. The PGL domain in the fusion protein, if functional, will then act on 6‐phosphogluconolactone to generate the end‐product 6‐phosphogluconate. We determined the functionality of 6PGL by demonstrating the formation of 6‐phosphogluconate. This assay is modified from a previous report describing the coupled assay [14].

In the first step, the purified G6PD‐6PGL fusion protein is added to the reaction buffer (100 mm Tris–HCl, pH 8) containing 0.2 mm NADP, 0.01 m MgCl_2_, 0.6 mm G6P, and the reaction of G6PD domain is confirmed (as monitored by NADP^+^ reduction at 340 nm at 25 °C) until saturation. The fusion protein is then removed by a microcon 10 kDa centrifugal filter from the reaction mixture. 200 μm NADP was added to the separated reaction mixture and incubated for 5 min to confirm that there was no leftover residual fusion enzyme as seen by a flat curve (no NADP reduction was seen as the G6PD‐6PGL enzyme was absent). One μg of 6 phosphogluconate dehydrogenase (6PGD) of *S. cerevisiae* obtained from Merck, Darmstadt, Germany (Cat No. P4553) was then added (6PGD acts on the end product of 6PGL and reduces NADP in the reaction mixture), and the activity of the 6PGD enzyme was detected through NADPH production at 340 nm. The functionality of the 6PGL domain was indirectly observed through 6PGD activity on the end product of the fusion protein (6‐phospho gluconate).

### HMG2 gene deletion in yeast

The HMG2 gene was deleted in the *hmg1*Δ deletion background by PCR‐mediated homologous recombination. A *hmg2*::LEU2 deletion cassette with flanking HMG2 regions on either end was generated by PCR using *hmg2*::*LEU2* del‐FP and *hmg2*::*LEU2* del‐RP and transformed into the *S. cerevisiae* BY4742 *hmg1*Δ strain. The *hmg1*Δ*hmg2*Δ double deleted strains were selected for leucine prototrophy on plates containing mevalonate (mevalonolactone, Cat No. M4667, Merck, Darmstadt, Germany) added at 5 mg·mL^−1^ from a stock of 330 mg·mL^−1^. The double deleted strain was confirmed by mevalonate auxotrophy since it has been earlier reported that double deletions of HMG1 and HMG2 show mevalonate auxotrophy [15].

### Estimation of total pools of NADP and NADPH

As NADPH pools are approximately 95% of the total pools of NADP plus NADPH, we have used the measurement of the total pools as reflective of the NADPH levels. For estimation of the total pools of NADP and NADPH, *zwf1Δ met15Δ S. cerevisiae* strains transformed with plasmids expressing G6PD, G6PD‐6PGL, G6PD‐tHMG, N403D, and S238Q under the TEF promoter and similarly transformed with the control vector were grown in SD medium containing 200 μm reduced GSH at 30 °C overnight and re‐inoculated in fresh SD medium at initial OD_600nm_ = 0.2; cells were allowed to grow at 30 °C till the early exponential growth phase OD_600nm_ = 0.6–0.8, with shaking at 220 rpm. An equal number of cells (OD_600nm_ = 1) were harvested at 2516 ***g*** and washed with sterile water, followed by resuspension of the cells in lysis buffer (100 mm KH_2_PO_4_, 1.2 m Sorbitol, pH 7). Spheroplasts were prepared by adding zymolase at the final concentration of 0.3 mg·mL^−1^ and subsequently incubating at 30 °C in a shaking incubator at 100 rpm for 1 h. A 100‐μL aliquot of these spheroplasts was mixed with an equal volume of the NADP/NADPH GloTM detection reagent from the NADP/NADPH‐GloTM assay kit (Cat No. G9081, Promega). The reaction mixture was incubated at room temperature for 45 min, and readings were taken using the POLARstar Omega luminescence reader. The data were analyzed using graphpad prism 5.0.

### Sclareol estimation

The yeast strains expressing the sclareol biosynthesis genes sclareol synthase and copal‐8‐ol diphosphate synthase, along with G6PD, G6PD‐6PGL, G6PD‐tHMG fusion proteins and the G6PD mutants were grown in SD medium overnight, shaking at 30 °C, then re‐inoculated at OD_600nm_ 0.02 into a 25 mL secondary culture, grown for 72 h, and 2.5 mL (10% v/v) of dodecane was added as an overlay and the culture was incubated for another 48 h, then centrifuged for 4930 ***g*** for 10 min, 1 mL of dodecane layer was collected and subjected to gas chromatography‐mass spectrometry (GC–MS) (Agilent 7890B GC,5977C MSD, Santa Clara, CA, USA). The HP5‐MS capillary column (30 m × 0.25 mm × 0.25 μm) was utilized in this analysis, and the carrier gas was helium (purity 99.999%). 1 μL was injected into a single‐mode inlet maintained at 320 °C. The GC oven was programmed for a temperature range of 100–320 °C at a ramp rate of 10 °C with a final hold of 10 min. The flow rate of helium was consistently maintained at 1.4 cm^2^·s^−1^. The sclareol peak was observed at the retention time of 13.25 min. Targeted peaks were identified by analyzing the mass spectra with the available NIST library and literature as described earlier [13]. The extracted ion chromatograms with ions of interest were analyzed (sclareol shows characteristic mass fragments of 177 191). A calibration curve over various concentration ranges made from the authentic standard (sclareol; Cat No. 515‐03‐7, Merck, Darmstadt, Germany) was used for the quantification.

### *In silico* studies of G6PD and mutants

The 3D model of G6PD was downloaded from the AlphaFold Protein Structure Database (https://alphafold.ebi.ac.uk/), and models of two mutants, N403D and S238QI239F, were generated computationally using the Maestro interface of Schrodinger. The Alphafold2 structure of *Sc*G6PD was used for these studies. All three structures were subjected to protein preparation [protein preparation wizard (PPW) of Maestro], where the structures were preprocessed by adding missing hydrogens, and appropriate bond orders were assigned to the structures. The protonation states of the polar residues were optimized with the protassign module of PPW, which uses PROPKA to predict pKa values (pH 7.0 ± 2.0) and side chain functional group orientations. The prepared structure was further used for the preparation of grids, molecular docking, and molecular dynamics (MD) simulations. The Glide [16] module of the Schrodinger suit was used to dock the substrates (G6P and NADP^+^) and the products (NADPH and 6PGL) to the three structures, thus obtaining six complexes of the wild‐type and mutant G6PD. All six complexes were energy minimized (for 5000 steepest descent steps) and subjected to implicit water MD simulations for 1 ns each, and the structures obtained after 1 ns simulations were considered for MM/GBSA (Molecular mechanics with generalized Born and surface area solvation) binding energy calculations using the Prime module of Schrodinger [17].

## Results

### Refining a genetic screen to isolate mutant enzymes that lead to increased NADPH levels

We examined previous genetic screens for NADPH levels to develop a robust and sensitive screen. In a screen for NADPH homeostatic genes previously developed in the lab, we used yeast cells depleted of glutathione [18]. The rationale for this screen was that the role of NADPH is generally masked by the presence of glutathione at millimolar (mm) concentrations relative to NADPH [present in micromolar (μm) concentrations] and that in low glutathione concentrations, NADPH levels become important, and thus genes affecting these levels can be identified. However, while it was reasonably successful in investigating the mitochondrial knockout collection [18], the screen was not sufficiently robust. Also, it lacked the required sensitivity for the current study.

Deletion of the G6PD encoding gene, *ZWF1*, in *S. cerevisiae* (*zwf1Δ*), leads to a distinct phenotype of methionine auxotrophy, which is known to result from NADPH deficiency. This deletion and its phenotype have previously been used to screen G6PD enzymes from different organisms [19]. However, our preliminary studies with this genetic background revealed that, though robust, it was not sufficiently sensitive in differentiating minor variations. Thus, to increase the sensitivity of the assay and refine the screen, we introduced a *met15Δ* deletion in the *zwf1Δ* background. Since *met15Δ* is an organic sulfur auxotroph, it needs organic sulfur. Thus, glutathione, a tripeptide containing cysteine, fulfills the organic sulfur auxotrophy. However, the strain, which is also a methionine auxotroph owing to *zwf1Δ*, faces an accentuated methionine requirement in this background, even with added glutathione.

**Fig. 2:**
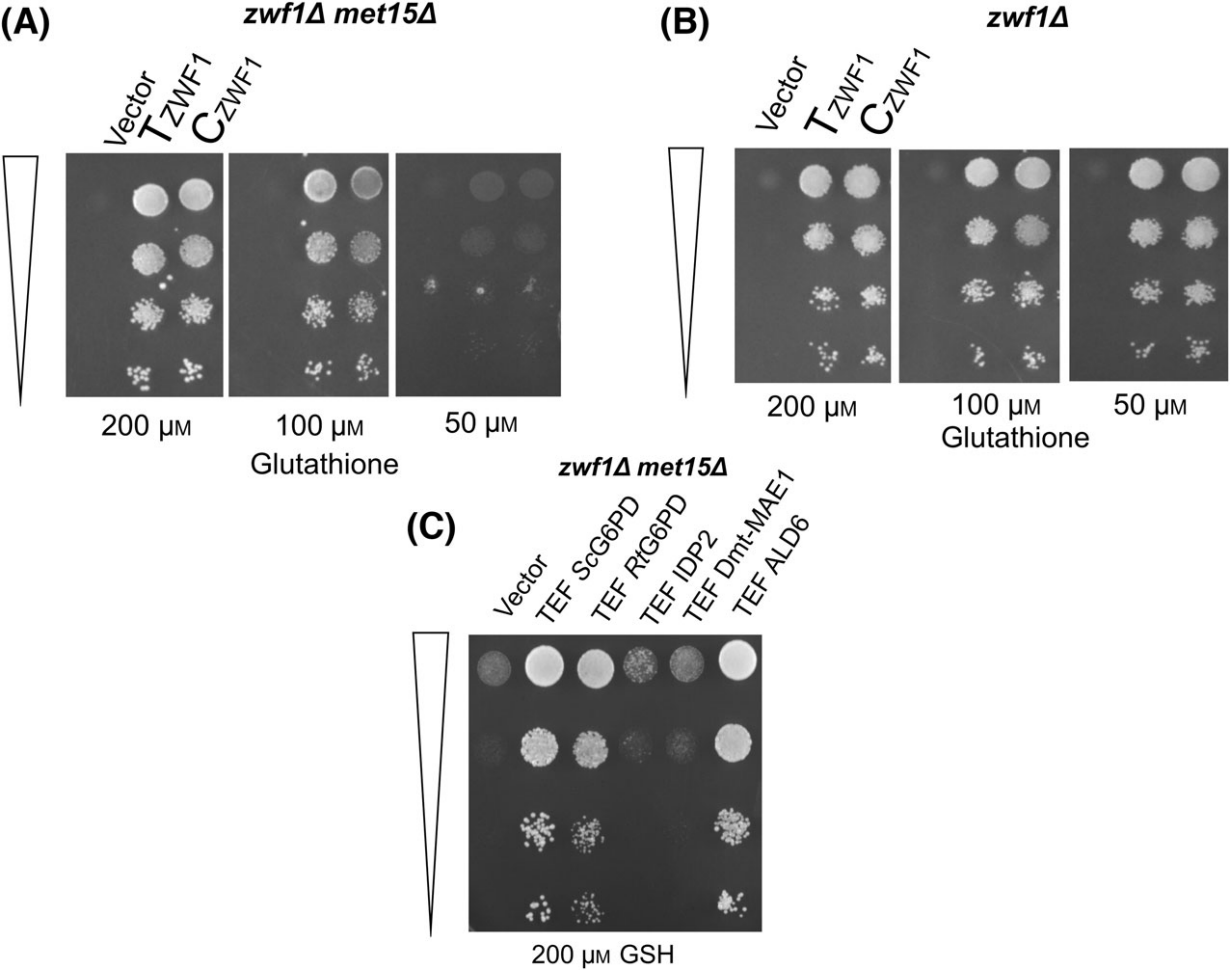
Comparison of *zwf1*Δ and *zwf1*Δ *met15*Δ as a screen for NADPH levels and evaluation of different NADPH‐generating enzymes. (A) *zwf1*Δ *met15*Δ and (B) *zwf1*Δ strains were each transformed with an empty vector, ZWF1 expressed under either the weak CYC promoter (C ZWF1) or the strong TEF promoter (T ZWF1), and serial dilutions spotted on different concentrations of glutathione plates. (C) Different NADPH‐generating enzymes in the cell, such as *Sc*G6PD, *Rt*G6PD, cytosolic isocitrate dehydrogenase (Idp2p), trunc‐malic enzyme, and cytosolic aldehyde dehydrogenase (Ald6p) were overexpressed in the *zwf1*Δ*met15*Δ strain under the TEF promoter, and dilution spotted along on plates containing glutathione. OD_600_ of dilution spots from top to bottom: 0.2, 0.02, 0.002, 0.0002. The experiments were repeated thrice, and this picture represents one of the experiments.

For preliminary evaluation of the screen, we decided to use the G6PD enzyme (encoded by *ZWF1* in *S. cerevisiae*) expressed from either a strong promoter (TEF) or a weak promoter (CYC). Differences in the behavior of these two clones on these screens would enable us to evaluate the screens. We thus evaluated TEF‐G6PD and CYC‐G6PD for the complementation of the methionine auxotrophy of the *zwf1Δ* and *zwf1Δ met15Δ* in both glutathione and methionine‐containing mediums across a range of concentrations. We observed that while the *zwf1Δ met15Δ* behaved similarly to the single *zwf1Δ* background in methionine medium, but, in glutathione medium over a narrow range of concentrations, we could see that the screen became more sensitive to minor differences such as those seen with the G6PD expressed under a strong or weak promoter (Fig. 2A,B). This genetic background has subsequently been used in all our screens and assays.

### G6PD is the most suitable enzyme choice for further improvement, as seen by the *zwf1*Δ *met15*Δ screen

Many enzymes in yeast are involved in contributing to the NADPH pools. To evaluate which of these enzymes might be most suited for intervention by mutagenesis or other strategies, we cloned the key enzymes known to be involved in playing a role in the cytosolic NADPH pools. We evaluated these enzymes by the *zwf1*Δ *met15*Δ assay. Thus, in addition to glucose 6‐phosphate dehydrogenase (*ZWF1*/G6PD), we also cloned the cytosolic aldehyde dehydrogenase enzyme (*ALD6*), the truncated malic enzyme (that was deleted for the 30 aa mitochondrial signal sequence) (t*MAE1*) and the cytosolic isocitrate dehydrogenase (*IDP2*). We observed that although Idp2p and tMae1p showed only weak complementation in this assay, the G6PD enzyme was able to confer significant growth (Fig. 2C). Thus, we considered it relevant to focus our efforts on this enzyme of the pentose phosphate pathway (PPP pathway). Ald6p also showed very good complementation (Fig. 2C). However, in a previous study, Ald6p overexpression alone did not lead to increased isoprenoid yields and was observed to decrease cell mass [20]. It increased isoprenoid production only when Ald6p overexpression was coupled with overexpression of a deregulated acetyl‐CoA synthetase (Acs1p). Further, in another study [21], *ALD6* downregulation (that resulted from the deletion of an upstream gene that overlapped with the *ALD6* promoter) led to decreased levels of isoprenoids. The decreased isoprenoids in this study resulted from multiple reasons. However, the results from these two studies suggested that *ALD6* might not be the best choice for the goal of the present study. Thus, we decided not to pursue it further.

The identification of G6PD (encoded by *ZWF1*) as an enzyme of choice for further enhancement underlined the importance of the pentose phosphate pathway, which has been the target of many investigators.

### A comparison of *Rt*G6PD and *Sc*G6PD shows that *Sc*G6PD has significantly higher activity than *Rt*G6PD

**Fig. 3:**
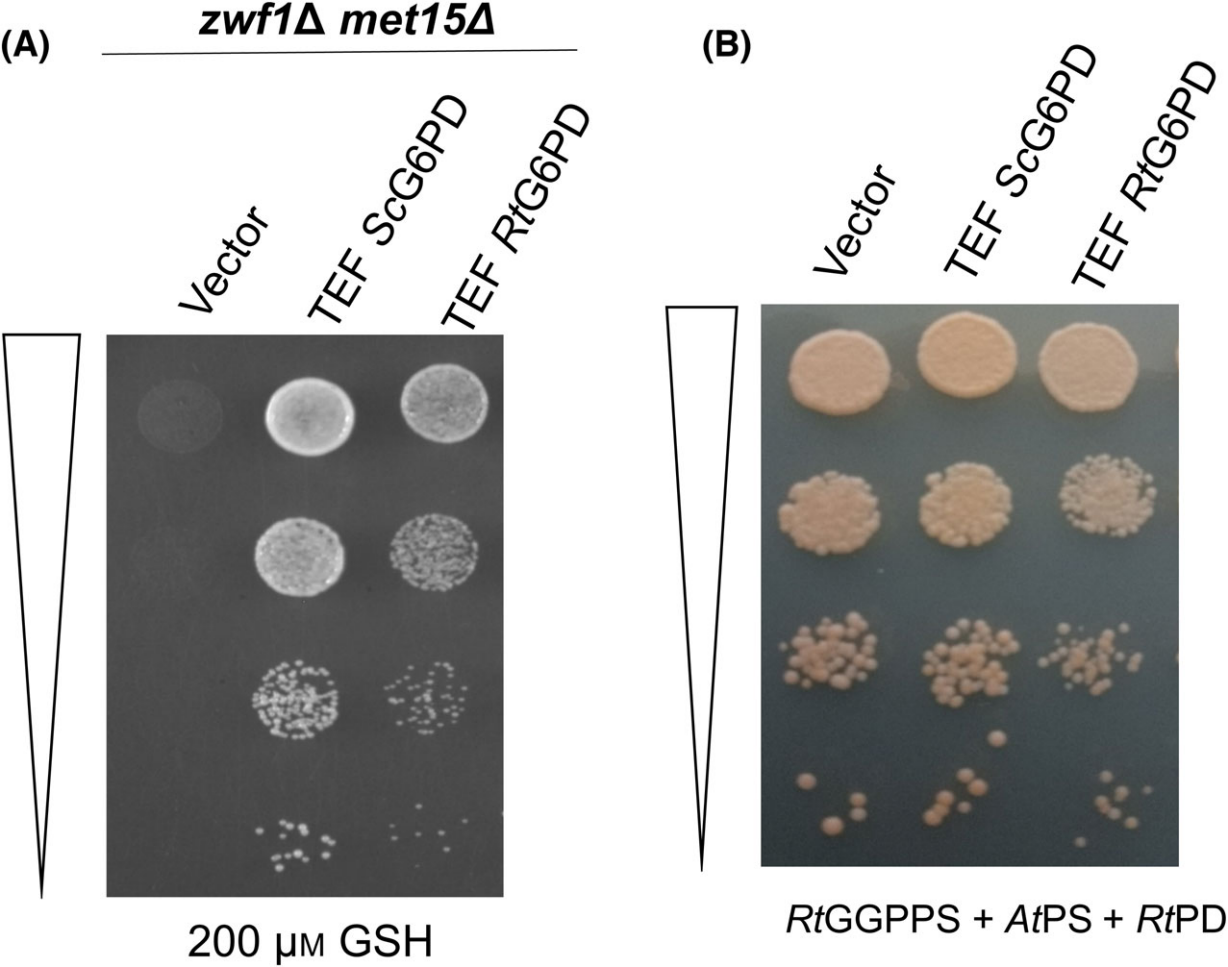
Comparison of *Sc*G6PD and *Rt*G6PD using the (A) complementation assay and (B) carotenoid pigmentation assay. For the complementation assay (A), *Sc*G6PD and *Rt*G6PD were expressed under the TEF promoter and transformed in *zwf1*Δ *met15*Δ, and serial dilutions were spotted on glutathione plates. For the carotenoid assay (B), *Sc*G6PD and *Rt*G6PD were expressed in a strain containing TEF *Rt*GGPPS (GGPP synthase), TEF *At*PS (Phytoene synthase), TEF *Rt*PD (Phytoene desaturase) and serial dilutions were spotted along with the empty vector control. OD_600_ of dilution spots from top to bottom: 0.2, 0.02, 0.002, 0.0002. Both experiments were performed in triplicate, and the image represents a single experiment.

G6PD seemed like the best choice for further improvement. However, would the G6PD from a carotenoid yeast be a better enzyme source? We decided to evaluate the glucose 6‐phosphate dehydrogenase enzyme from the red yeast *Rhodosporidium toruloides* (*Rt*G6PD). Since *R. toruloides* is an oleaginous and carotenogenic yeast and is known to be the highest producer of carotenoids among yeasts [22], we considered that it might have a higher requirement of NADPH and therefore that the enzyme from this yeast might be superior to the *S. cerevisiae* enzyme. Thus, the cDNA encoding *Rt*G6PD (which is GC rich and has multiple introns) was custom synthesized after codon optimization (Accession no. OQ291226) and expressed downstream of the TEF promoter. This gene was also evaluated in the *zwf1Δ* met15*Δ* screen. Surprisingly, *Rt*G6PD conferred less growth than *Sc*G6PD in this screen (Fig. 3A).

The use of the *zwf1*Δ screen by a previous group for comparing G6PD across species suggested that the *in vivo* results did not always tally with the *in vitro* activities [19]. Although we have used a more stringent screen (using *zwf1Δ met15Δ* rather than only *zwf1Δ*), we decided further confirmation was required. Therefore, we also evaluated this by examining the effect on carotenoid pigmentation. We transformed the genes encoding the carotenogenic enzymes of *R. toruloides* GGPP synthase (*RtGGPPS*), phytoene dehydrogenase (*RtPD*), and phytoene synthase of *A. thaliana* (*AtPS*) into *S. cerevisiae*. These strains are able to make red pigment, and the use of the mono‐functional *At*PS restricted the carotenoids to lycopene, making it more sensitive (M. Wadhwa and A. K. Bachhawat, unpublished observations). However, the *Rt*G6PD could not improve the pigmentation levels over the *Sc*G6PD ability (Fig. 3B). The possibility still existed that the *in vivo* data might not entirely reflect the actual *in vitro* properties since protein stability could also contribute to *in vivo* activities. We thus purified the two enzymes from *E. coli* using His‐tagged proteins that we purified by Ni‐NTA columns (Fig. S1). Both enzymes showed optimum activity at pH 8 (Fig. S2), and their specific activities were compared at pH 8. We found that *Rt*G6PD had significantly lower activity (11 ± 0.9 units per mg protein) compared to *Sc*G6PD (92.5 ± 2.2 units per mg protein). Although surprising, it also confirmed what was observed in the *in vivo* assay. This suggested that the *Sc*G6PD enzyme was better suited than *Rt*G6PD for further improvement.

### Evaluation of G6PD fusions to 6 Phosphogluconolactonase (6PGL) as a possible strategy for enhancing NADPH production efficiency

Fusion enzymes of consecutive enzymes in a pathway form synthetic metabolons and are an important strategy for cells to have more efficient processes. The substrate channeling from the product of a previous enzyme allows for more efficient catalysis. Further, the physical proximity of one enzyme's product to the substrate channel of another enzyme enhances efficiency [14].

In some parasitic protozoans, such as plasmodium species and other protozoans where NADPH requirements are essential, interestingly, one observes that two of the enzymes of the pentose phosphate pathway are found as fusion enzymes [23, 24] and differ in some of their kinetic parameters. Therefore, we sought to explore whether such a fusion might be more effective in an organism (such as yeasts) where such a fusion does not naturally occur. To identify which type of fusions might be best suited for enzymes in the pathway, we studied the different fusions existing in nature [23].

**Fig. 4:**
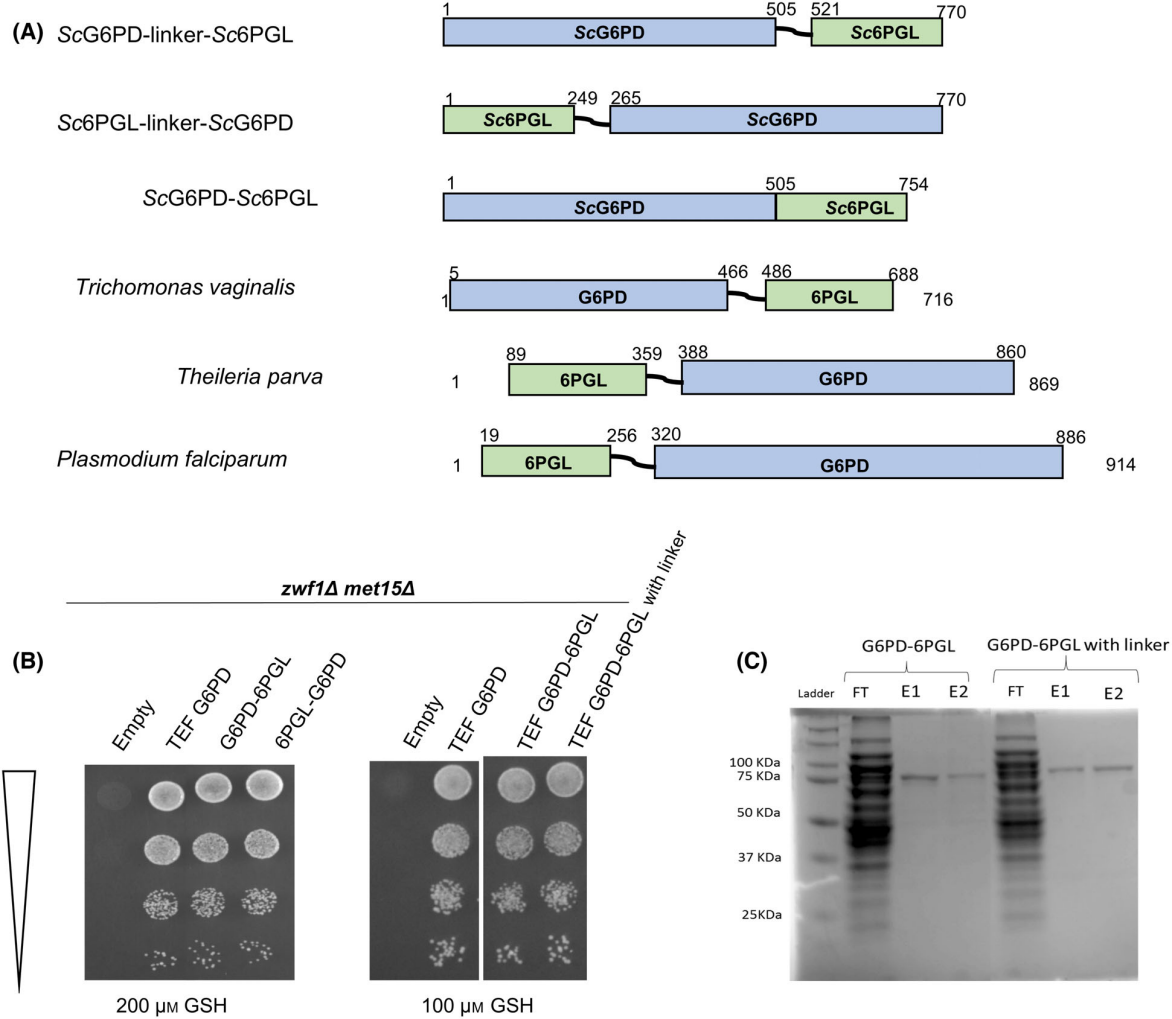
Pictorial representation of G6PD‐6PGL fusion proteins and the *in vivo* functionality of their G6PD domains. (A) Schematic representation of the gene fusions created and their comparison with naturally occurring fusions. The fusion proteins made in this study aligned with the existing fusion proteins in *T. vaginalis*, *T. parva*, and *P. falciparum*. Gene fusions of G6PD and 6PGL (B) evaluated by complementation of *zwf1Δmet1*5*Δ* and (C) their purification profiles were displayed on 12% SDS/PAGE.

Although there are three enzymes in the oxidative part of the pentose phosphate pathway, which include glucose 6‐p dehydrogenase (G6PD encoded by *ZWF1*), 6‐phosphogluconolactonase (6PGL encoded by *SOL3*), and 6‐phosphogluconate dehydrogenase (6PGD encoded by *GND1*), the naturally occurring gene fusions occurred only between the first and second enzymes. However, the orientations were different in different organisms. Based on this comparative investigation, we created fusions of G6PD and 6PGL with and without a 16‐aa linker between the two enzymes (Fig. 4A). We created them as G6PD‐L‐6PGL, 6PGL‐L‐G6PD, and a G6PD‐6PGL (without a linker). We first evaluated them in the genetic screen (*zwf1Δmet15Δ*). The growth conferred seemed quite comparable to the growth conferred by the control *Sc*G6PD, indicating that at least the G6PD enzyme was still functioning well (Fig. 4B). However, it was important to determine whether each domain was functional and the kinetic parameters. We cloned these His‐tagged proteins in the pET23a expression vector and expressed them in *E. coli*. Of the various fusions, only the G6PD‐L‐6PGL and G6PD‐6PGL could be purified (Fig. 4C), and thus we proceeded with these enzymes.

The first task was determining whether the individual G6PD and 6PGL domains were functional. The G6PD activity was confirmed *in vitro* using the assay for G6PD enzymes, where NADPH formation is measured. As expected, we could detect G6PD activity *in vitro* as well. To determine if the 6PGL region (encoding 6‐phosphogluconolactonase activity) was functional, we initially performed the assay for G6PD to generate 6‐phosphogluconolactone, the substrate for 6‐PGL. The 6‐PGL activity was then demonstrated using the product of this enzymatic reaction and coupling it to the next enzyme, 6‐phosphogluconate dehydrogenase (6PGD), which was procured from a commercial source and can be measured by NADPH detection assays (Fig. S3). After establishing the functionality of both domains, the activity was compared with the *Sc*G6PD enzyme. Activity measurements of the linker‐containing enzyme indicated a less efficient enzyme (37 ± 0.8 units per mg protein). In contrast, without a linker, the fusion activity was significantly higher (93.9 ± 1.1 units per mg protein), though the activity was a little less than the single G6PD domain protein alone (141.5 ± 3.5 units per mg protein) (Table 1).

**Table 1:** Specific activities of *Sc*G6PD fusion proteins and mutants.

	Sp. activity (units per mg protein)^a^
*Sc*G6PD	141.5 ± 3.5
G6PD‐6PGL	93.9 ± 1.1
G6PD‐L‐6PGL	37 ± 0.8
H161R	153 ± 2.5
R226P	89.5 ± 2.8
S238E I239F	132 ± 5.5
N403D	188 ± 1.9
S238Q I239F	155.5 ± 1.5
S238Q I239F N403D	189 ± 14
H161R S238Q I239F N403D	168 ± 9

^a^ Specific activities were obtained from at least two independent purifications each, with at least three technical replicates of enzyme measurements. ± designate the standard deviations. One unit of the enzyme reduces 1 μmol of NADP per min.

**Fig. 5:**
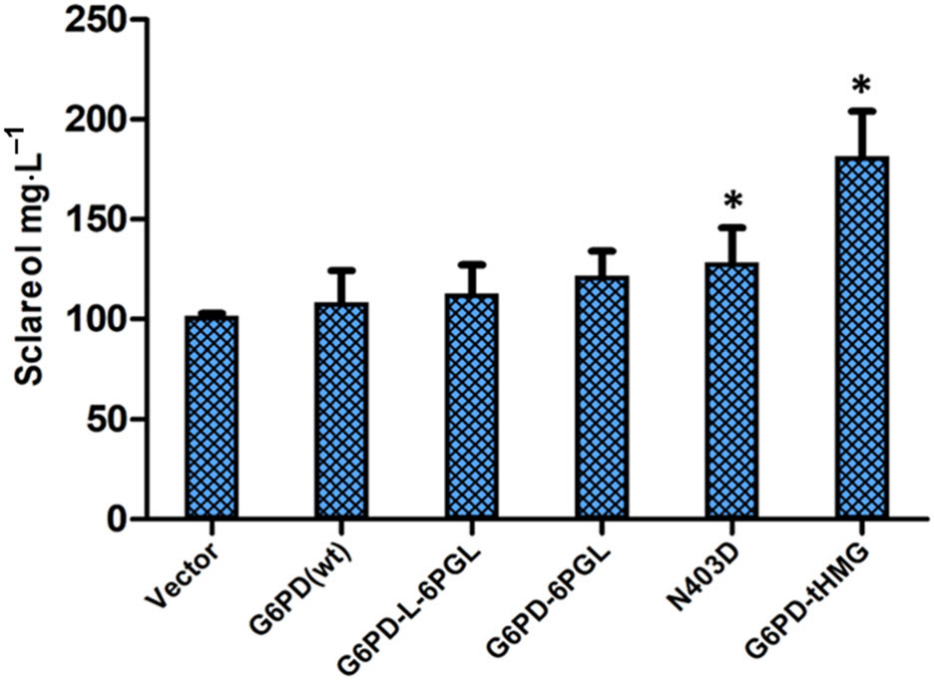
Estimation of sclareol yields in strains overexpressing wild‐type G6PD, G6PD‐6PGL, G6PD‐tHMG fusion proteins, and G6PD N403D mutant. Sclareol levels were estimated in the strains containing sclareol biosynthesis genes. overexpressed with G6PD, G6PD‐6PGL, G6PD‐6PGL with linker, G6PD‐tHMG and N403D mutant by GC–MS analysis. The graph shows a representative data set of three biological replicates. Error bars indicate standard deviation. Asterisk (*) indicates statistically significant differences (*T*‐test, *P*‐value < 0.05) relative to G6PD (wt).

Investigation of the kinetic parameters of the *Plasmodium falciparum*, *Plasmodium vivax*, and *Giardia lamblia* enzymes had earlier revealed that although the fusion showed comparable activity, the *K*_m_ towards G6P was improved [14, 24, 25]. Therefore, we sought to investigate the affinity of the fusions towards the primary substrates, G6P and NADP. Kinetic measurements were carried out with the linker‐containing fusion and the linker‐less fusion. Interestingly, we observed that in the more active linker‐less fusion, the affinity towards NADP was lower, but the affinity towards G6P was surprisingly almost five times higher (Table 2). This indicated that the pattern of this synthetically created fused enzyme was similar to what is seen in the natural enzymes of *Plasmodium falciparum*, *Plasmodium vivax*, and *Giardia lamblia*; and suggested that there was a possibility that this fusion might be able to perform well *in vivo*, where G6P could be in short supply. Therefore, we considered it essential to evaluate the impact of these fusions on the production of the diterpenoid sclareol. Genes for this diterpenoid were expressed in yeasts along with *Rt*GGPP synthase and either WT G6PD or the fusion enzyme, and sclareol was estimated as mentioned in the methods. The experiment was carried out in five replicates, and the three best producers were compared. We observed that these fusions showed a 12–18% increase over the WT gene, with the fusion without the linker showing higher sclareol levels between the two (Fig. 5).

**Table 2:** Steady‐state *K*_m_ values of G6PD^a^, fusion proteins, and mutants for Glucose 6‐phosphate and NADP^+^.

	G6PD	G6PD‐6PGL	G6PD‐L‐6PGL	N403D	S238Q
G6P (μm)	2.6 ± 0.3	0.6 ± 0.0	2.3 ± 0.2	1.0 ± 0.0	7.6 ± 0.5
NADP^+^ (μm)	0.6 ± 0.1	2.6 ± 0.4	5.6 ± 0.2	0.8 ± 0.1	1.1 ± 0.1

^a^ *K*_m_ values were obtained from at least two independent purifications each, with at least two technical replicates of enzyme measurements. ± designate the standard deviations.

### Construction of a novel synthetic metabolon, with G6PD fused to tHMG1

**Fig. 6:**
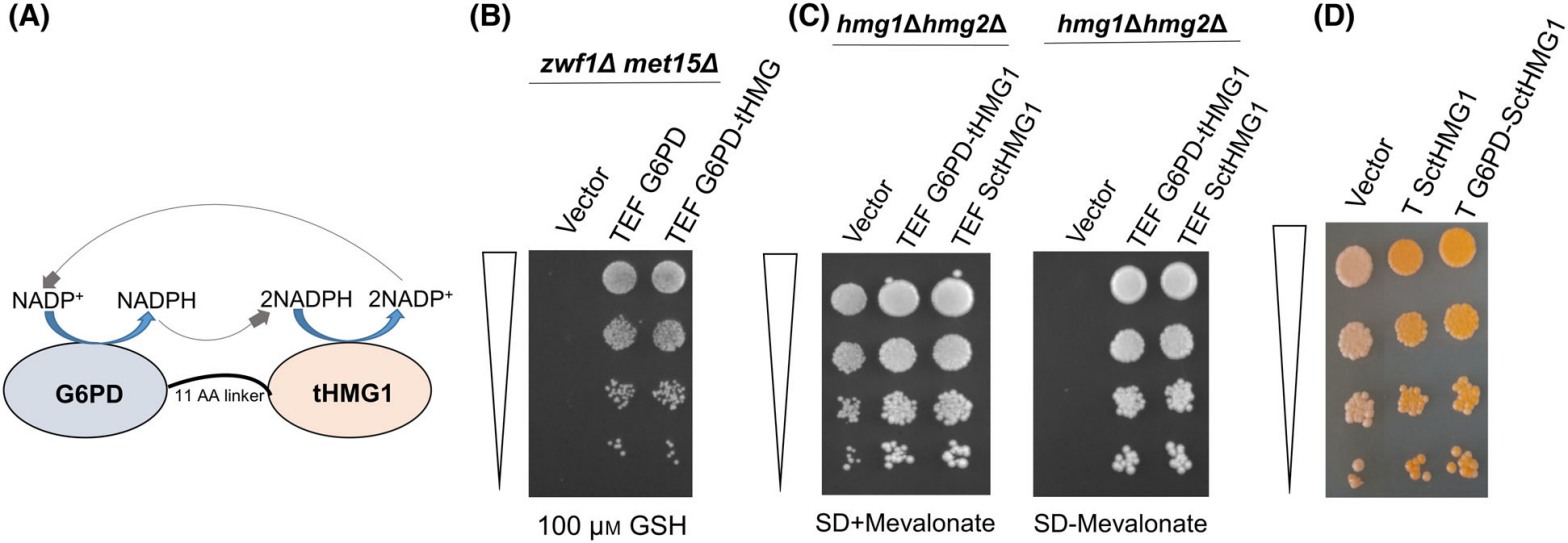
G6PD‐tHMG1 fusion protein and its functionality. Schematic, along with the functional activity of the individual domains and the fusion. The G6PD‐tHMG1 fusion is schematically represented (A). (B, C) evaluation of the functionality of the G6PD domain and the tHMG1 domains, and (D) the functionality of the fusion evaluated using the carotenoid assay (as explained in the methods) using a WT strain containing *Rt*GGPPS, *At*PS, and *Rt*PD genes and compared with control tHMG1, a known flux enhancer. Details in materials and methods. OD_600_ of dilution spots from top to bottom: 0.2, 0.02, 0.002, 0.0002.

Although the G6PD‐6PGL and 6PGL‐G6PD fusions of *S. cerevisiae* were constructed based on insights on similar naturally occurring fusions occurring in parasitic protozoans, we also considered the creation of a fusion previously not found in nature. In the fusion design, we fused the NADPH‐generating enzyme *Sc*G6PD with an enzyme of the isoprenoid pathway, 3‐hydroxy‐3‐methyl‐glutaryl‐coenzyme A reductase (HMG‐CoA reductase), that uses NADPH. The expectation was that these could be more efficient in utilizing NADPH for the enzymatic reaction. Based on this rationale, we constructed a fusion of *Sc*G6PD with the truncated HMG‐CoA reductase, tHmg1p (Fig. 6A). The truncated Hmg1p (tHmg1p) lacks the N‐terminal regulatory region of *HMG1*, thereby preventing its feedback regulation [26, 27]. Since this was a fusion not known to exist in nature, we first needed to examine if the two domains were functional. To do so, we evaluated the ability of the fusion to complement the *zwf1Δ met15Δ* mutant and the ability of the fusion protein to complement the mevalonate auxotrophy of the *hmg1Δ hmg2Δ* mutant [15]. Using these *in vivo* functional assays, we found that both domains were functional as seen by the complementation, and the functionality was comparable to the WT G6PD (Fig. 6B) and WT tHmg1p, respectively (Fig. 6C), as seen by the *in vivo* assay. The pigmentation levels in the carotenoid‐producing strain overexpressed with the G6PD‐*Sc*tHmg1 protein hinted at its efficiency in increasing the isoprenoid flux (Fig. 6D).

This novel G6PD‐tHMG fusion under the TEF promoter showed about 1.8‐fold more sclareol production than the WT G6PD (Fig. 5).

### Rational mutagenesis of the *Sc*G6PD based on evolutionary and structural insights identifies mutant proteins with superior activity

The G6PD enzyme is highly conserved across evolution. Although naturally occurring mutants have been detected with lower activity [28], increased activity mutants have not been reported. However, activity measurements in native enzymes across species suggest wide variation, and it might be possible to identify residues that would lead to increased activity upon mutagenesis.

To identify such residues that might be potentially able to increase catalytic activity, we utilized the Hotspot Wizard 3.0 server. Hotspot Wizard 3.0 is an online server (https://loschmidt.chemi.muni.cz/hotspotwizard/) that can create mutation libraries and detect residue alterations of a protein structure [29] that can affect the protein's stability or catalytic properties. From the presented residues, one can look at tolerated substitutions based on sequence homologs of 200 sequences. We carried out the analysis using the alphafold2 predicted structure. As an additional criterion, we also focused on residues that fell in the catalytic pockets but, were non‐essential for catalysis.

Six active site pocket residues were identified accordingly (Table S4). These were H161, R226, N403, S238, and I239. Each residue was changed to the most conserved residue among the homologs. The specific mutations we made were decided based on the permissible substitutions that could preserve the protein's function. Thus, H161 was mutated to H161R (R being the most conserved residue at that position). Similarly, R226 was mutated to P, N403 to N403D, S238 to S238Q, and I239 to I239F. We also created a double mutant, S238EI239F. The mutants were made by splice overlap extension PCR. We initially verified them by the yeast screen, and this was done by expressing them under a weak promoter, examining them in the *zwf1Δmet15Δ* screen (Fig. S4), and then cloning into an *E. coli* expression vector with a His‐tag. The mutants were purified by Ni‐NTA, and then their activity was evaluated.

Interestingly, we found that although the R226P mutants showed lower activity, the single mutants H161R and S238Q showed almost 10–15% higher activity, and the double mutant S238Q I239F also showed an approximately 10% increase in activity. More interestingly, the N403D mutant showed an almost 25% increase in catalytic activity (Table 1).

To see if combining these superior mutants in a single protein would increase protein enzyme activity, we introduced the separate mutations in a single clone and evaluated the activity of the triple mutant. Thus, in one case, we generated H161R S238QI239F N403D, which showed an approximately 15% increase in activity over the wild‐type but did not increase the activity beyond the single mutant N403D and was, in fact, a little lower than the N403D activity alone. The mutant S238QI239F N403D, which was also created, showed activity comparable to N403D, which was significantly higher than the wild type, but the combined mutations did not lead to any significant synergistic increase (Table 1).

We also evaluated the relative NADPH levels of the mutants *in vivo* since activity measurements *in vitro* do not consider the protein's stability *in vivo*. We evaluated these mutants using an *in vivo* assay kit that measures the total pools of NADPH and NADP. However, since NAPDH pools reflect ~ 95% of the pools, they largely reflect the NADPH pools. This can be seen from the estimations done in a *zwf1Δ* background. As expected, the *zwf1*Δ cells had significantly lower levels of NADPH that could be restored by expressing the *ZWF1* gene under the TEF promoter. The mutant S238QI239F also showed levels comparable to the WT clone. However, using this assay, the N403D mutant seemed to show slightly higher levels of NADPH (Fig. S5), but the assay itself might not be sensitive enough to differentiate between the variants significantly.

To evaluate how effective, the N403D mutant would be relative to WT *Sc*G6PD *in vivo* in an engineered system making heterologous isoprenoids, we compared the formation of the diterpenoid sclareol with the WT and N403D mutant as described earlier and in the methods. We observed that the sclareol yields in the strain expressing the N403D mutant were 15–25% higher when compared with the wild‐type G6PD (Fig. 5). The N403D hyperactive mutant was also fused to tHmgp to see if it might improve the sclareol yields further. However, the N403D‐tHMG fusion did not show enhanced sclareol yields compared to the G6PD‐tHMG fusion protein (Fig. S6).

### Investigations into the structural basis of the increased activity of the hyperactive mutants

G6PD catalyzes the rate‐limiting step of the oxidative pentose‐phosphate pathway. It converts D‐glucose 6‐phosphate (G6P) and NADP^+^ to 6‐phospho‐D glucono‐1‐5 lactone (6PGL), NADPH, and H^+^. Six complexes were modeled where the substrates (G6P and NADP^+^) and products (6PGL and NADPH) bound to the wild type, N403D, and S238QI239F mutants of G6PD. Energy minimization and implicit water molecular dynamic simulations were carried out for 1 ns each. The structures obtained after 1 ns simulations were considered to calculate the molecular mechanics generalized Born surface area (MM/GBSA) binding energy of the substrate and ligands with G6PD. A comparative analysis of these binding energies was done in quest of a possible explanation for the higher activity of the two mutants N403D and S238QI239F relative to the WT type at the atomistic level (Fig. S7). From the total binding energy values (MM/GBSA dG Bind), it was found that the binding of both products (NADPH and 6PGL) weakened in both mutants as compared to the wild type (Table S5). Even the binding of NADP^+^ weakens in both mutants as compared to the wild‐type, but the binding of G6P (substrate) becomes stronger in the case of mutants as compared to the wild‐type (Table S6). This correlates with the experimentally determined *K*_m_ values of N403D (but not with the S238QI239F), showing that the N403D mutant indeed shows stronger binding to substrate G6P than the wild‐type (Table 2). The MM/GBSA binding energy (dG Bind) values in the case of products show that the binding of both products (NADPH and 6PGL) weakens in both mutants as compared to the wild type (Table S5), indicating the inhibition by these products on the protein might be less or these products might be easily released after the reaction in mutants compared to the wild type.

## Discussion

The need for enhancing NADPH levels in metabolic pathways such as the isoprenoid pathway to overproduce heterologous isoprenoids is well recognized. In this study, we have taken an approach that could be more cost‐efficient for the cell than the existing approaches. This is achieved through the use of catalytically superior variants of the key NADPH‐generating enzymes. When we scanned the literature for natural mutant variants that were superior in activity, we found that in humans, several mutants were reported for G6PD, but these were all showing lower activity [30]. Only one mutant had higher activity, but investigations revealed that it was a regulatory mutant and only affected the expression but not the intrinsic enzyme activity [31]. In *Plasmodium* spp. several natural variants were also investigated, but none showed significantly higher activity [32]. From a synthetic biology perspective, the strategic approach to improve the enzyme itself (through either mutagenesis or fusions, etc.) does not seem to have been attempted so far, and this is what was investigated in this study.

While evaluating different NADPH‐generating enzymes in the genetic screen that we developed, we also evaluated the G6PD enzyme from the carotenogenic yeast *R. toruloides*. A surprising finding, however, was that the *R. toruloides* G6PD enzyme was almost 9‐fold less efficient than the *S. cerevisiae* enzyme. This was completely unexpected, as it was thought that the higher NADPH requirements of *R. toruloides* during carotenoid production might be met by a more efficient *Rt*G6PD enzyme. One possible explanation is that there are other important sources of NADPH in *R. toruloides*. Alternatively, as *R. toruloides* makes carotenoids, which are capable of tackling certain reactive oxygen species, perhaps the need for excess NADPH in that organism is not as one would expect.

We created synthetic metabolons through gene fusions using the *S. cerevisiae* G6PD enzyme. In the initial design of these synthetic metabolons, we mimicked nature in some of the designs (where the first enzymes of the PPP were fused). The G6PD‐6PGL fusion proteins were based on similar fusions seen in protozoan parasites that must combat significant oxidative stress in host cells. As active G6PD enzymes are known to be dimeric in nature, we were not sure how synthetic fusions would behave. Therefore, we made fusions in both orientations, with or without a linker. Both orientations yielded functional enzymes, that were interestingly comparable in activity with the WT enzyme, as seen in the *in vivo* assays. Fusions of these two sequential enzymes of the PPP pathway are seen in several parasites, where these pathways are so important that they have also been explored as therapeutic targets [32, 33]. Interestingly, these fusions also seem useful in the context of synthetic biology, which also requires higher NADPH levels. Although the G6PD domain in the synthetic G6PD‐6PGL metabolons constructed were found to be less active than the individual G6PD domains alone *in vitro* and *in vivo*, the possibility existed that they could improve the NADPH pools in the cell by channeling the product directly to the substrate binding pocket of the second enzyme [34]. Further, although the affinity for NADP^+^ was lower in the fusion, it showed a higher affinity to the other substrate, G6P, than the individual G6PD enzyme (Table 2). This might improve the functioning of the enzyme under specific conditions, such as glucose limitation. The overall effectiveness of these fusions relative to WT was evaluated *in vivo* through the production of the diterpenoid sclareol, and marginally higher sclareol levels were observed.

Overproduction of truncated HMG1 (tHmg1p) is well known to increase the flux in the isoprenoid pathway. But it has also been reported that the yields can be further increased by overexpression of an enzyme that enhances NADPH levels [3]. However, we have opted for a novel approach of fusing tHmg1p with a strong NADPH producer, G6PD. The G6PD‐tHmg1p fusion enzyme compared well with *Sc*tHmg1p alone, a known flux enhancer in the isoprenoid pathway, and compared well with the WT G6PD in the carotenoid assay. This suggested that the fusion was likely to be at least as effective as the parent enzyme. When evaluated through the production of the diterpenoid sclareol, we observed that sclareol estimations were significantly higher in the fusion compared to the G6PD enzyme (since tHmg1p alone can also increase yields); it is clear that this fusion also has the potential for further development and improvement. The sclareol assays were done using a 4‐plasmid system, which might account for some of the variability. The use of integrated strains is also likely to result in higher yields.

In rational mutagenesis, we exploited an online server (Hotspot Wizard) [29] to help identify potential residues, but then additional constraints were imposed for deciding which mutants would be created experimentally. This algorithm has been successfully used to make enzymes with increased activity for industrial purposes [35, 36], and thus we sought to use it in this study as well. The strategy proved immensely successful because three out of the five mutants we created showed higher catalytic activity. Among these, N403D consistently showed improved activity *in vitro* and increased sclareol levels compared with the wild‐type enzyme. The *in silico* MD simulation studies have suggested a lower binding affinity of the products to the pocket. Thus, this may facilitate the release of these products and thereby enhance the activity. Further improvement by combining the mutations did not occur, but with the single N403D alone, we were able to obtain some increase in the production of the diterpenoid sclareol, which suggests that the mutant is not just effective *in vitro* but *in vivo* as well.

In conclusion, in a genetic screen that we developed, we evaluated different metabolic enzymes generating NADPH and found that glucose‐6‐phosphate dehydrogenase was the best and most suited for further optimization. Multiple approaches were taken to improve the characteristics of the enzyme from *S. cerevisiae*. In a synthetic metabolon approach, we have created and evaluated G6PD fusions with the downstream PPP enzyme and also G6PD fusions with tHmg1p, a key enzyme in the mevalonate pathway that is a major consumer of NADPH in the pathway. In addition, we have taken a rational mutagenesis approach that has yielded new mutants that show increased activity. Thus, both approaches yielded new variants that were beneficial for isoprenoid production. While demonstrating these for the diterpenoid sclareol, we believe they could generally apply to other terpenoids.

## Conflict of interest

Sri Harsha Adusumilli declares that he has no conflict of interest. Anuthariq Alikkam Veetil declares that he has no conflict of interest. Chinmayee Choudhury declares that she has no conflict of interest. Banani Chattopadhyaya declares that she has no conflict of interest. Diptimayee Behera declares that she has no conflict of interest. Anand Kumar Bachhawat declares that he has no conflict of interest.

## Author contributions

SHA: conceptualization, validation, methodology, formal analysis, investigation, data curation, writing original draft; AAV: cloning and *in vitro* activities of some G6PD mutants; AKB: conceptualization, supervision, formal analysis, funding acquisition, project administration, writing including review and editing; CC: *in silico* analysis, MD simulations of G6PD and its mutants with substrates and products; BC: sclareol experiments including cloning, extraction of sclareol from yeast cells, analysis of sclareol data, editing; DB: GC–MS analysis and estimation of sclareol samples.

## Supporting information


**Table S1.** List of strains used in the study.
**Table S2.** List of primers used in the study.
**Table S3.** List of plasmids used in the study.
**Table S4.** The Hotspot Wizard data of the selected residues for mutagenesis.
**Table S5.** Binding energies and their components of the 6‐phospho‐D‐glucono lactone (6‐PDGL) and NADP^+^ with wild‐type G6PD and mutants.
**Table S6.** Binding energies and their components of the substrate and NADP^+^ with wild‐type G6PD and mutants.
**Fig. S1.** Purification profiles of *Sc*G6PD and *Rt*G6PD.
**Fig. S2.** Effect of pH on the activity of *Sc*G6PD and *Rt*G6PD.
**Fig. S3.** 6PGL activities of the fusion proteins without and with a linker.
**Fig. S4.** *In vivo* functionality of G6PD mutants.
**Fig. S5.** Estimation of total NADPH and NADP pools *in vivo*.
**Fig. S6.** Estimation of sclareol yields in strains overexpressing G6PD‐tHMG and G6PD N403D mutant–tHMG fusion proteins.
**Fig. S7.** MM/GBSA Binding energies of the substrate (G6P and NADP^+^) and product (NADPH and 6PGL) molecules with the wild type (WT) and mutant (S238QI239F and N403D) G6PD structures.

## Data Availability

All data generated or analyzed during this study are included in this published article [and its supplementary information files].

## References

[1] Kwak S , Yun EJ , Lane S , Oh EJ , Kim KH and Jin YS (2020) Redirection of the glycolytic flux enhances isoprenoid production in *Saccharomyces cerevisiae* . Biotechnol J 15, 1–10. 10.1002/biot.201900173 31466140

[2] Scalcinati G , Partow S , Siewers V , Schalk M , Daviet L and Nielsen J (2012) Combined metabolic engineering of precursor and cofactor supply to increase α ‐santalene production by *Saccharomyces cerevisiae* . Microb Cell Fact 11, 117. 22938570 10.1186/1475-2859-11-117 PMC3527295

[3] Paramasivan K and Mutturi S (2017) Regeneration of NADPH coupled with HMG‐CoA reductase activity increases squalene synthesis in *Saccharomyces cerevisiae* . J Agric Food Chem 65, 8162–8170. 28845666 10.1021/acs.jafc.7b02945

[4] Oh Y , Lee T , Lee S , Oh E , Ryu Y , Kim M and Seo J (2007) Dual modulation of glucose 6‐phosphate metabolism to increase NADPH‐dependent xylitol production in recombinant *Saccharomyces cerevisiae* . J Mol Catal B 47, 37–42.

[5] Zhao X , Shi F and Zhan W (2015) Overexpression of ZWF1 and POS5 improves carotenoid biosynthesis in recombinant *Saccharomyces cerevisiae* . Lett Appl Microbiol 61, 354–360. 26179622 10.1111/lam.12463

[6] Hong J , Park SH , Kim S , Kim SW and Hahn JS (2019) Efficient production of lycopene in *Saccharomyces cerevisiae* by enzyme engineering and increasing membrane flexibility and NAPDH production. Appl Microbiol Biotechnol 103, 211–223. 30343427 10.1007/s00253-018-9449-8

[7] Liu H , Wang F , Deng L and Xu P (2020) Genetic and bioprocess engineering to improve squalene production in *Yarrowia lipolytica* . Bioresour Technol 317, 123991. 32805480 10.1016/j.biortech.2020.123991 PMC7561614

[8] Li T , Liu GS , Zhou W , Jiang M , Ren YH , Tao XY , Liu M , Zhao M , Wang FQ , Gao B *et al*. (2020) Metabolic engineering of *Saccharomyces cerevisiae* to overproduce squalene. J Agric Food Chem 68, 2132–2138. 31989819 10.1021/acs.jafc.9b07419

[9] Mannazzu I , Landolfo S , da Silva TL and Buzzini P (2015) Red yeasts and carotenoid production: outlining a future for non‐conventional yeasts of biotechnological interest. World J Microbiol Biotechnol 31, 1665–1673. 26335057 10.1007/s11274-015-1927-x

[10] Gietz RD , Schiestl RH , Willems AR and Woods RA (1995) Studies on the transformation of intact yeast cells by the LiAc/SS‐DNA/PEG procedure. Yeast 11, 355–360. 7785336 10.1002/yea.320110408

[11] Sambrook J , Fritsch EF and Maniatis T (1989) Molecular Cloning: A Laboratory Manual. Vol. 3. 2nd edn. Cold Spring Harbor Laboratory Press, Cold Spring Harbor, NY.

[12] Mumberg D , Müller R and Funk M (1995) Yeast vectors for the controlled expression of heterologous proteins in different genetic backgrounds. Gene 156, 119–122. 7737504 10.1016/0378-1119(95)00037-7

[13] Yadav R , Chattopadhyay B , Kiran R , Yadav A , Bachhawat AK and Patil SA (2022) Microbial electrosynthesis from carbon dioxide feedstock linked to yeast growth for the production of high‐value isoprenoids. Bioresour Technol 363, 127906. 36087648 10.1016/j.biortech.2022.127906

[14] Morales‐Luna L , González‐Valdez A , Hernández‐Ochoa B , Arreguin‐Espinosa R , Ortega‐Cuellar D , Castillo‐Rodríguez RA , Martínez‐Rosas V , Cárdenas‐Rodríguez N , Enríquez‐Flores S , Canseco‐ávila LM *et al*. (2021) Glucose‐6‐phosphate dehydrogenase::6‐phosphogluconolactonase from the parasite *Giardia lamblia*. A molecular and biochemical perspective of a fused enzyme. Microorganisms 9, 1678. 34442758 10.3390/microorganisms9081678 PMC8399836

[15] Basson ME , Thorsness M , Finer‐Moore J , Stroud RM and Rine J (1988) Structural and functional conservation between yeast and human 3‐hydroxy‐3‐methylglutaryl coenzyme a reductases, the rate‐limiting enzyme of sterol biosynthesis. Mol Cell Biol 8, 3797–3808. 3065625 10.1128/mcb.8.9.3797-3808.1988 PMC365438

[16] Friesner RA , Murphy RB , Repasky MP , Frye LL , Greenwood JR , Halgren TA , Sanschagrin PC and Mainz DT (2006) Extra precision glide: docking and scoring incorporating a model of hydrophobic enclosure for protein‐ligand complexes. J Med Chem 49, 6177–6196. 17034125 10.1021/jm051256o

[17] Bowers KJ , Chow E , Xu H , Dror RO , Eastwood MP , Gregersen BA , Klepeis JL , Kolossvary I , Moraes MA , Sacerdoti FD *et al*. (2006) Scalable algorithms for molecular dynamics simulations on commodity clusters. *Proceedings of the 2006 ACM/IEEE Conference on Supercomputing*, 43.

[18] Yadav S , Mody TA , Sharma A and Bachhawat AK (2020) A genetic screen to identify genes influencing the secondary redox couple NADPH/NADP^+^ in the yeast *Saccharomyces cerevisiae* . G3 (Bethesda) 10, 371–378. 31757928 10.1534/g3.119.400606 PMC6945034

[19] Heinisch JJ , Knuesting J and Scheibe R (2020) Investigation of heterologously expressed glucose‐6‐phosphate dehydrogenase genes in a yeast zwf1 deletion. Microorganisms 8, 546. 32283834 10.3390/microorganisms8040546 PMC7232176

[20] Shiba Y , Paradise EM , Kirby J , Ro DK and Keasling JD (2007) Engineering of the pyruvate dehydrogenase bypass in *Saccharomyces cerevisiae* for high‐level production of isoprenoids. Metab Eng 9, 160–168. 17196416 10.1016/j.ymben.2006.10.005

[21] Chen Y , Wang Y , Liu M , Qu J , Yao M , Li B , Ding M , Liu H , Xiao W and Yuan Y (2019) Primary and secondary metabolic effects of a key gene deletion (Δ*YPL062W*) in metabolically engineered Terpenoid‐producing *Saccharomyces cerevisiae* . Appl Environ Microbiol 85, e01990‐18. 30683746 10.1128/AEM.01990-18 PMC6585493

[22] Mata‐Gómez LC , Montañez JC , Méndez‐Zavala A and Aguilar CN (2014) Biotechnological production of carotenoids by yeasts: an overview. Microb Cell Fact 13, 12. 24443802 10.1186/1475-2859-13-12 PMC3922794

[23] Stover NA , Dixon TA and Cavalcanti ARO (2011) Multiple independent fusions of Glucose‐6‐phosphate dehydrogenase with enzymes in the pentose phosphate pathway. PloS One 6, e22269. 21829610 10.1371/journal.pone.0022269 PMC3148214

[24] Clarke JL , Scopes DA , Sodeinde O and Mason PJ (2001) Glucose‐6‐phosphate dehydrogenase‐6 phosphogluconolactonase. A novel bifunctional enzyme in malaria parasites. Eur J Biochem 268, 2013–2019. 11277923 10.1046/j.1432-1327.2001.02078.x

[25] Jortzik E , Mailu BM , Preuss J , Fischer M , Bode L , Rahlfs S and Becker K (2011) Glucose‐6‐phosphate dehydrogenase – 6‐phosphogluconolactonase: a unique bifunctional enzyme from *Plasmodium falciparum* . Biochem J 436, 641–650. 21443518 10.1042/BJ20110170

[26] Dimster‐Denk D , Thorsness MK and Rine J (1994) Feedback regulation of 3‐hydroxy‐3‐methylglutaryl coenzyme a reductase in *Saccharomyces cerevisiae* . Mol Biol Cell 5, 655–665. 7949422 10.1091/mbc.5.6.655 PMC301081

[27] Polakowski T and Stahl U (1998) Overexpression of a cytosolic hydroxymethylglutaryl‐CoA reductase leads to squalene accumulation in yeast. Appl Microbiol Biotechnol 49, 66–71. 9487712 10.1007/s002530051138

[28] Yoshida A and Beutler E (1978) Human glucose‐6‐phosphate dehydrogenase variants: a supplementary tabulation. Ann Hum Genet 41, 347–355. 626479 10.1111/j.1469-1809.1978.tb01902.x

[29] Sumbalova L , Stourac J , Martinek T , Bednar D and Damborsky J (2018) HotSpot wizard 3.0: web server for automated design of mutations and smart libraries based on sequence input information. Nucleic Acids Res 46, W356–W362. 29796670 10.1093/nar/gky417 PMC6030891

[30] Yoshida A , Beutler E and Motulsky AG (1971) Human Glucose‐6‐phosphate dehydrogenase variants. Bull World Health Organ 45, 243–253. 5316621 PMC2427914

[31] Yoshida A (1970) Amino acid substitution (histidine to tyrosine) in a glucose‐6‐phosphate dehydrogenase variant (G6PD Hektoen) associated with over‐production. J Mol Biol 52, 483–490. 5492291 10.1016/0022-2836(70)90414-6

[32] Haeussler K , Berneburg I , Jortzik E , Hahn J , Rahbari M , Schulz N , Preuss J , Zapol'skii VA , Bode L , Pinkerton AB *et al*. (2019) Glucose 6‐phosphate dehydrogenase 6‐phosphogluconolactonase: characterization of the *Plasmodium vivax* enzyme and inhibitor studies. Malar J 18, 22. 30683097 10.1186/s12936-019-2651-z PMC6346587

[33] Preuss J , Jortzik E and Becker K (2012) Glucose‐6‐phosphate metabolism in *Plasmodium falciparum* . IUBMB Life 64, 603–611. 22639416 10.1002/iub.1047

[34] Camagna M , Grundmann A , Bar C , Koschmieder J , Beyer P and Welsch R (2019) Enzyme fusion removes competition for geranylgeranyl diphosphate in carotenogenesis. Plant Physiol 179, 1013–1027. 30309967 10.1104/pp.18.01026 PMC6393812

[35] Luo X , Wang Y , Zheng W , Sun X , Hu G , Yin L , Zhang Y , Yin F and Fu Y (2022) Simultaneous improvement of the thermostability and activity of lactic dehydrogenase from *Lactobacillus rossiae* through rational design. RSC Adv 12, 33251–33259. 36425200 10.1039/d2ra05599f PMC9677063

[36] Guo X , An Y , Chai C , Sang J and Jiang L (2020) Bioresource technology construction of the R17L mutant of Mt C1LPMO for improved lignocellulosic biomass conversion by rational point mutation and investigation of the mechanism by molecular dynamics simulations. Bioresour Technol 317, 124024. 32836036 10.1016/j.biortech.2020.124024

