# Supplementary material for: Glucose 6‐phosphate dehydrogenase variants increase NADPH pools for yeast isoprenoid production
Source: FEBS Open Bio. 2024 Jan 2;14(3):410–25. doi: 10.1002/2211-5463.13755 (PMC10909971; doi:10.1002/2211-5463.13755)
Supplement: Supplementary file 1 — Table S1. List of strains used in the study. Table S2. List of primers used in the study. Table S3. List of plasmids used in the study. Table S4. The Hotspot Wizard data of the selected residues for mutagenesis. Table S5. Binding energies and their components of the 6‐phospho‐D‐glucono lactone (6‐PDGL) and NADP+ with wild‐type G6PD and mutants. Table S6. Binding energies and their components of the substrate and NADP+ with wild‐type G6PD and mutants. Fig. S1. Purification profiles of ScG6PD and RtG6PD. Fig. S2. Effect of pH on the activity of ScG6PD and RtG6PD. Fig. S3. 6PGL activities of the fusion proteins without and with a linker. Fig. S4. In vivo functionality of G6PD mutants. Fig. S5. Estimation of total NADPH and NADP pools in vivo. Fig. S6. Estimation of sclareol yields in strains overexpressing G6PD‐tHMG and G6PD N403D mutant–tHMG fusion proteins. Fig. S7. MM/GBSA Binding energies of the substrate (G6P and NADP+) and product (NADPH and 6PGL) molecules with the wild type (WT) and mutant (S238QI239F and N403D) G6PD structures. [file FEB4-14-410-s001.docx]

**Supplementary information**

**Glucose 6-phosphate dehydrogenase variants increase NADPH pools for yeast isoprenoid production**

**Sri Harsha Adusumilli^1^, Anuthariq Alikkam Veetil^1,#^, Chinmayee Choudhury^2^, Banani Chattopadhyaya^1^, Diptimayee Behera^3^ and Anand Kumar Bachhawat^1*^**

**^1^** Department of Biological Sciences, Indian Institute of Science Education and Research Mohali, S.A.S. Nagar, Punjab, India

**^2^** Department of Experimental Medicine and Biotechnology, PGIMER, Chandigarh, India

**^3^**Department of Earth and Environmental Sciences, Indian Institute of Science Education and Research Mohali, S.A.S. Nagar, Punjab, India

**^#^** Current address Department of Chemistry and Biomedical Sciences, Linnaeus university, Universitetsplatsen 1, 392 31 Kalmar, Sweden

*Corresponding author: E-mail: anand@iisermohali.ac.in, anand.bachhawat@gmail.com

**Table S1. List of strains used in the study**

|  | **Strains** | **Genotype** | **Source** |
| --- | --- | --- | --- |
| 1 | Δ*zwf1*  (BY4741 Background) | MATa; *his*3Δ1; *leu*2Δ0; *met*15Δ0; *ura*3Δ0; *zwf1*::kanMX4 | Euroscarf  (Y01971) |
| 2 | Δ*zwf1*  (BY4742 Background) | MATα; *his*3Δ1; *leu*2Δ0; *lys*2Δ0; *ura*3Δ0; *zwf1*::kanMX4 | Euroscarf  (Y11971) |
| 3 | ABC276 | MATα; *his*3Δ1; *leu*2Δ0; *lys*2Δ0; *ura*3Δ0; *trp1*Δ0GAL | Lab strain  (S288c derived) |
| 4 | *E.coli* DH5α | F^–^ φ80*lac*ZΔM15 Δ(*lac*ZYA-*arg*F)U169 *rec*A1 *end*A1 *hsd*R17(r_K_^–^, m_K_^+^) *pho*A *sup*E44 λ^–^*thi*-1 *gyr*A96 *rel*A1 | Novagen |
| 5 | *E.coli* BL21(DE3)pLysS | F^–^*omp*T *hsd*S_B_ (r_B_^–^, m_B_^–^) *gal dcm*(DE3) pLysS(Cam^R^) | Novagen |
| 6 | Δ*hmg1*  (BY4742 background) | MATα; *his*3Δ1; *leu*2Δ0; *lys*2Δ0; *ura*3Δ0; YML075c::kanMX4 | Euroscarf  (Y16733) |
| 7 | Δ*hmg1Δhmg2*  (BY4742 background) | MATα; *his*3Δ1; *lys*2Δ0; *ura*3Δ0; Δ*hmg*1::kanMX4; Δ*hmg*2::LEU2 | This study |

**Table S2. List of primers used in the study**

| **Primer name** | **Primer sequence (5’ 🡪 3’)** |
| --- | --- |
| trunc MAE1 BamHI-FP | ACTCGGATCCATGTGGCCTATTCAGCAATC |
| trunc MAE1 EcoRI-RP | ACTTGAATTCCTACAATTGGTTGGTGTGC |
| IDP2 BamHI-FP | ATTCGGATCCATGACAAAGATTAAGGTAGC |
| IDP2 SalI-RP | TCTAGTCGACTTACAATGCAGCTGCCTCG |
| ALD6 BamHI-FP | TCATGGATCCATGACTAAGCTACACTTTGACAC |
| ALD6 XhoI-RP | TACTCTCGAGTTACAACTTAATTCTGACAG |
| *Sc*G6PD BamHI-FP | TCATGGATCCATGAGTGAAGGCCCCGTCAAATTCG |
| *Sc*G6PD XhoI-RP | TACTCTCGAGCTAATTATCCTTCGTATC |
| 6PGL BamHI-FP | ACTAGGATCCGAGATCTTTGACTTGAAAAACG |
| 6PGL SalI-RP | ATCTGTCGACCTAAAAAGTTTTCGTTTG |
| G6PD-6PGL FP | GCCAGAAGATACGAAGGATAATGTGACAGTCGGTGTGTTTTCTG |
| G6PD-6PGL RP | CAGAAAACACACCGACTGTCACATTATCCTTCGTATCTTCTGGC |
| G6PD-link-6PGL FP | GCCAGAAGATACGAAGGATAATTTGGATATAGGCGTTGGGATGATTAAAAGGAACAAAAAGAATCTGGCTCAAGTGACAGTCGGTGTGTTTTCTG |
| G6PD-link-6PGL RP | CAGAAAACACACCGACTGTCACTTGAGCCAGATTCTTTTTGTTCCTTTTAATCATCCCAACGCCTATATCCAAATTATCCTTCGTATCTTCTGGC |
| *Sc*G6PD-link-*Sc*tHMG link FP | TAAGCCAGATACGAAGGATAATGGTGGCGGTGGTAGCGGCGGTGGCGGCAGCGGTATGGACCAATTGGTGAAAACTGAAG |
| *Sc*G6PD-link-*Sc*tHMG link RP | CTTCAGTTTTCACCAATTGGTCCATACCGATGCCGCCACCGCCGCTACCACCGCCACCATTATCCTTCGTATCTTCTGGCTTA |
| *Sc*tHMG1- XhoI RP | TACTCTCGAGTTAGGATTTAATGCAGGTGACG |
| *Sc*G6PD NheI-FP | TCATGCTAGCATGAGTGAAGGCCCCGTCAAATTC |
| *Rt*G6PD NheI-FP | TCTAGCTAGCATGTCTGGTGTTGCCGCAGCCTC |
| *Rt*G6PD 6X-HIS SalI-RP | TCTAGTCGACTCAGTGGTGATGGTGATGATGCAAGACGGATGTTGAAGGCC |
| H161R FP | TAATCGTAGAGAAACCTTTCGGCCGTGACCTGGCCTCTGCCAGGGAGCT |
| H161R RP | AGCTCCCTGGCAGAGGCCAGGTCACGGCCGAAAGGTTTCTCTACGATTA |
| R226P FP | GCGTTCAGATTTCGTTTAAAGAGCCTTTCGGCACCGAAGGCCGTGGCGG |
| R226P RP | CCGCCACGGCCTTCGGTGCCGAAAGGCTCTTTAAACGAAATCTGAACGC |
| S238E I239F FP | AAGGCCGTGGCGGCTATTTCGACGAATTTGGCATAATCAGAGACGTGATGCAGAAC |
| S238E I23F RP | GTTCTGCATCACGTCTCTGATTATGCCAAATTCGTCGAAATAGCCGCCACGGCCTT |
| N403D FP | ATGCTACCCAAGTCACAGATCTGGATCTAACTTACGCAAGCAGGTACCA |
| N403D RP | TGGTACCTGCTTGCGTAAGTTAGATCCAGATCTGTGACTTGGGTAGCAT |
| K171 VRY FP | TGGCCTCTGCCAGGGAGCTGCAAVRYAACCTGGGGCCCCTCTTTAAAGAAG |
| K171 RYB RP | CTTCTTTAAAGAGGGGCCCCAGGTTRYBTTGCAGCTCCCTGGCAGAGGCCA |
| S238E/Q FP | AAGGCCGTGGCGGCTATTTCGACSAATTTGGCATAATCAGAGACGTGAT |
| S238E/Q RP | ATCACGTCTCTGATTATGCCAAATTSGTCGAAATAGCCGCCACGGCCTT |
| M486 TWY FP | ATATGCAAAAACACAAGTATGTTTWYCCCGAAAAGCACCCTTACGCTTGGC |
| M486 TWY RP | GCCAAGCGTAAGGGTGCTTTTCGGGRWAAACATACTTGTGTTTTTGCATAT |
| *hmg2*::LEU2 del-FP | ATGTCACTTCCCTTAAAAACGATAGTACATTTGGTAAAGCTTAAGCAAGGATTTTCTTAA |
| *hmg2*::LEU2 del-RP | TTATAATAATGCTGAGGTTTTACAGGGGGGCCCTTTGTTAGTGGGAATACTCAGGTATC |

**Table S3. List of plasmids used in the study**

| **Plasmids** | **Description** | **Source** |
| --- | --- | --- |
| pRS315TEF-*Rt*GGPPS | GGPP synthase from *R.toruloides* (ATCC 204091) custom synthesized and cloned in pRS315TEF | Lab strain  (Wadhwa and Bachhawat 2016) |
| pRS416TEF-*At*-PS | Phytoene synthase from *A.thaliana* from TAIR(clone U16445) cloned without chloroplast signal into pRS416TEF | Lab strain |
| pRS314TEF-*Rt*-PD | Phytoene desaturase from *R.toruloides* (ATCC 204091) custom synthesized and cloned in PRS314TEF | Lab strain  (Wadhwa and Bachhawat 2016) |
| pRS313TEF-IDP2 | IDP2 cloned in PRS313TEF between *Bam*HI and *Sal*I sites | This study |
| pRS313TEF-tMAE1 | The first 90bps of MAE1 truncated and cloned between *Bam*HI and *Sal*I in pRS313TEF | This study |
| pRS313TEF-ALD6 | ALD6 cloned in pRS313TEF between *Bam*HI and *Xho*I sites | This study |
| pRS313TEF-*Sc*G6PD | *Sc*G6PD cloned in pRS313TEF between *Bam*HI and *Eco*RI sites | This study |
| pRS313TEF-*Rt*G6PD | *Rt*G6PD custom synthesized and cloned in pRS313TEF between *Bam*HI and *Xho*I sites | This study  (Accession no: OQ291226) |
| pRS313CYC-*Sc*G6PD | *Sc*G6PD cloned in pRS313CYC between *Bam*HI and *Xho*I sites | This study |
| pRS313TEF-*Sc*G6PD-*Sc*6PGL with linker | *Sc*G6PD N term-*Sc*6PGL C term fused with 16 AA linker and cloned in pRS313TEF between *Bam*HI and *Xho*I sites | This study |
| pRS313TEF-*Sc*6PGL-*Sc*G6PD with linker | *Sc*6PGL N- term-*Sc*G6PD C-term fused with 16 AA linker and cloned in pRS313TEF between *Bam*HI and *Xho*I sites | This study |
| pRS313TEF-*Sc*G6PD-*Sc*6PGL | *Sc*G6PD Nterm-*Sc*6PGL C-term fused without linker and cloned in pRS313TEF between *Bam*HI and *Xho*I sites | This study |
| pRS313TEF-*Sc*G6PD-*Sc*tHMG | *Sc*G6PD and *Sc*tHMG1 linked with a 7AA Gly-Ser linker and cloned *Bam*HI and *Eco*RI | This study |
| pRS313CYC-G6PD H161R | *Sc*G6PD H161R cloned in pRS313CYC between *Bam*HI and *Xho*I sites | This study |
| pRS313CYC-G6PD R226P | *Sc*G6PD R226P cloned in pRS313CYC between *Bam*HI and *Xho*I sites | This study |
| pRS313CYC-G6PD S238EI239F | *Sc*G6PD S238EI239F cloned in pRS313CYC between *Bam*HI and *Xho*I sites | This study |
| pRS313CYC-G6PD N403D | *Sc*G6PD N403D cloned in PRS313CYC between *Bam*HI and *Xho*I sites | This study |
| pRS313CYC-G6PD S238QI239F | *Sc*G6PD S238QI239F cloned in pRS313CYC between *Bam*HI and *Xho*I sites | This study |
| PET23a-*Sc*G6PD-6XHIS | *Sc*G6PD 6XHIS tagged and cloned in PET23a between *Nhe*I and *Sal*I sites | This study |
| PET23a-*Rt*G6PD-6XHIS | *Rt*G6PD 6XHIS tagged and cloned in PET23a between *Nhe*I and *Sal*I sites | This study |
| PET23a- *Sc*G6PD-*Sc*6PGL with linker-6XHIS | *Sc*G6PD-*Sc*6PGL with linker 6XHIS tagged and cloned in PET23a between *Nhe*I and *Sal*I sites | This study |
| PET23a- *Sc*6PGL-*Sc*G6PD with linker 6XHIS | *Sc*6PGL-*Sc*G6PD with linker 6XHIS tagged and cloned in PET23a between *Nhe*I and *Sal*I sites | This study |
| PET23a- *Sc*G6PD-*Sc*6PGL -6XHIS | *Sc*G6PD-*Sc*6PGL 6XHIS tagged and cloned in PET23a between *Nhe*I and *Sal*I sites | This study |
| PET23a- G6PD H161R -6XHIS | *Sc*G6PD H161R 6XHIS tagged and cloned in PET23a between *Nhe*I and *Sal*I sites | This study |
| PET23a- G6PD R226P -6XHIS | G6PD R226P 6XHIS tagged and cloned in PET23a between *Nhe*I and *Sal*I sites | This study |
| PET23a- G6PD S238QI239F 6XHIS | *Sc*G6PD S238QI239F 6XHIS tagged and cloned in PET23a between *Nhe*I and *Sal*I sites | This study |
| PET23a- G6PD N403D 6XHIS | *Sc*G6PD N403D 6XHIS tagged and cloned in PET23a between *Nhe*I and *Sal*I sites | This study |
| PET23a- G6PD S238QI239F 6XHIS | *Sc*G6PD S238QI239F 6XHIS tagged and cloned in PET23a between *Nhe*I and *Sal*I sites | This study |
| PET23a- G6PD M486Y 6XHIS | *Sc*G6PD M486Y 6XHIS tagged and cloned in PET23a between *Nhe*I and *Sal*I sites | This study |
| PET23a- G6PD N403D 6XHIS | *Sc*G6PD N403D 6XHIS tagged and cloned in PET23a between *Nhe*I and *Sal*I sites | This study |
| pRS314TEF-*Cc*CLS | Custom synthesized copal-8-ol diphosphate synthase of *Cistus creticus* cloned under TEF promoter in pRS314TEF | (Yadav et al. 2022) |
| pRS416TEF-*Ss*SS | Custom synthesized sclareol synthase of *Salvia sclerea* cloned under TEF promoter in p416TEF | (Yadav et al. 2022) |

**Table S4. The Hotspot Wizard data of the selected residues for mutagenesis**

| **Residue** | **Sec structure** | **SASA**  **(RSA)** | **M**  **score** | **Pockets** | **Tunnel** | **Av.**  **B-factor** |
| --- | --- | --- | --- | --- | --- | --- |
| **H161** | Bend | 103.83A^2^ (53.5%) | 6 | Catalytic  13 | 1 | 93.90 A^2^ |
| **R226** | Bend | 164.36A^2^ (68.2%) | 6 | ---- | ----- | 97.06 A^2^ |
| **S238** | H-bonded turn | 81.78A^2^ (67%) | 7 | Catalytic  4 | 1 | 90.57 A^2^ |
| **I239** | H-bonded turn | 34.01A^2^ (18.7%) | 6 | Catalytic  4,12 | 1 | 93.49 A^2^ |
| **N403** | Extended strand | 74.14A^2^ (46.9%) | 5 | 23 | ----- | 93.96 A^2^ |
| **M486** | Loop | 103.83A^2^ (53.5%) | 4 | 3,11,36 | ------ | 85.75 A^2^ |

**Table S5. Binding energies and their components of the 6-phospho-D-glucono lactone (6-PDGL) and NADP^+^ with wild type G6PD and mutants**

|  | **MMGBSA dG Bind** | **MMGBSA dG Bind Coulomb** | **MMGBSA dG Bind Hbond** | **MMGBSA dG Bind Lipo** | **MMGBSA dG Bind vdW** |
| --- | --- | --- | --- | --- | --- |
| **G6PD (WT)-NADP** | -61.1 | -44.0 | -17.4 | -6.4 | -41.6 |
| **G6PD(S238QI239F)-NADP^+^** | -51.0 | -41.3 | -16.6 | -6.3 | -42.6 |
| **G6PD (N403D)-NADP** | -46.3 | 15.9 | -15.0 | -3.4 | -34.9 |
| **G6PD (WT)-PDGL** | -19.5 | 9.4 | -8.0 | -4.9 | -7.5 |
| **G6PD(S238QI239F)-NADP^+^** | -18.1 | 19.7 | -7.7 | -0.5 | -9.0 |
| **G6PD (N403D)-PDGL** | -16.1 | 55.8 | -5.4 | -3.6 | -15.0 |

**Table S6.** **Binding energies and their components of the substrate and NADP^+^ with wild type G6PD and mutants**

|  | **MMGBSA dG Bind** | **MMGBSA dG Bind Coulomb** | **MMGBSA dG Bind H-bond** | **MMGBSA dG Bind Lipo** | **MMGBSA dG Bind vdW** |
| --- | --- | --- | --- | --- | --- |
| **G6PD (WT)-NADP^+^** | -96.8 | 4.4 | -18.7 | -12.6 | -56.1 |
| **G6PD(S238QI239F)- NADP^+^** | -76.3 | -37.4 | -15.6 | -9.6 | -55.0 |
| **G6PD (N403D)-NADP^+^** | -83.7 | 1.9 | -17.2 | -8.4 | -49.7 |
| **G6PD (WT)-G6P** | -13.2 | 30.5 | -6.9 | -0.9 | -11.2 |
| **G6PD(S238QI239F)-G6P** | -17.1 | 9.3 | -7.5 | -1.4 | -9.3 |
| **G6PD (N403D)-G6P** | -16.1 | 44.9 | -6.8 | -2.8 | -14.9 |


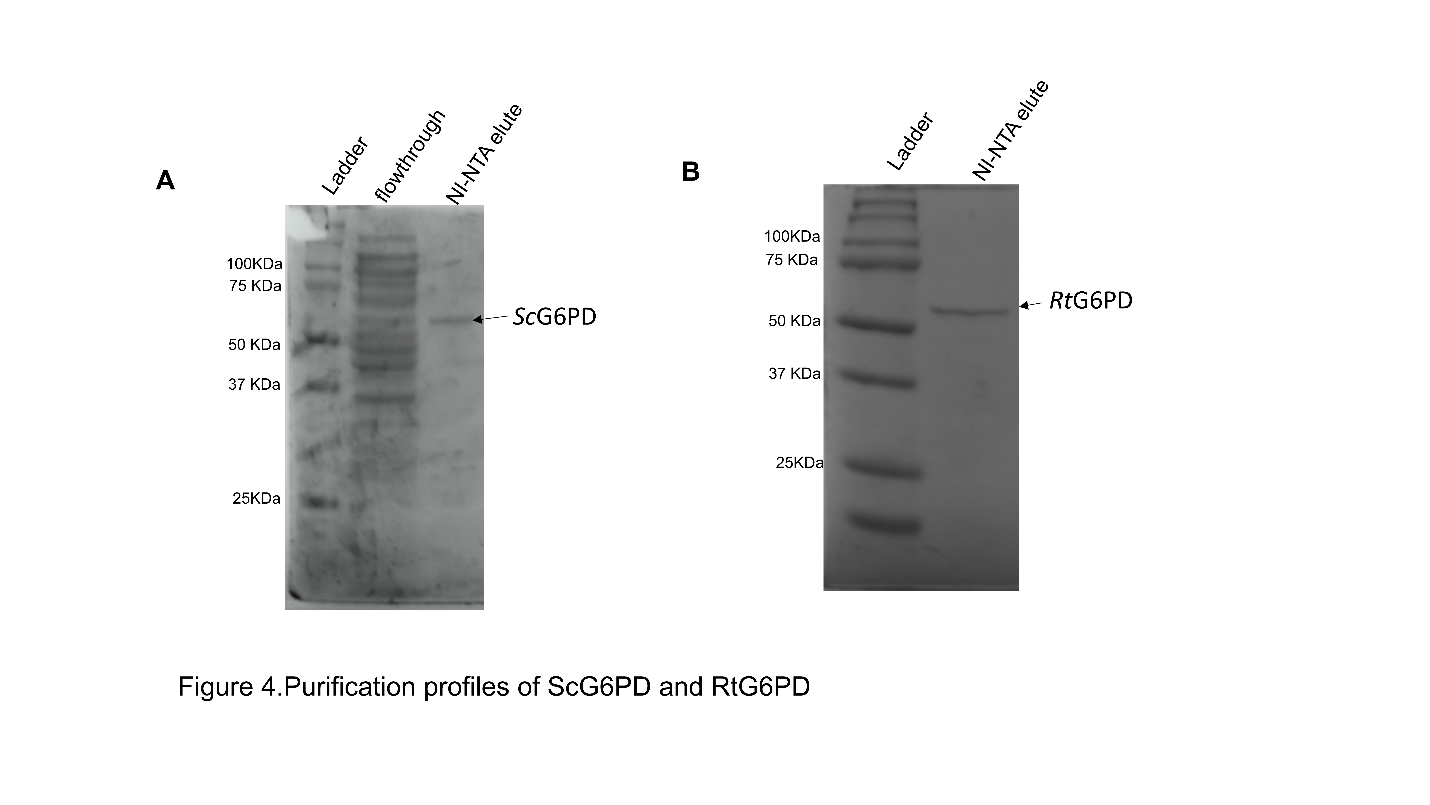


**Figure S1. Purification profiles of ScG6PD and RtG6PD.**  SDS-PAGE (12%) showing the Ni-NTA purified (A) ScG6PD and (B) RtG6PD


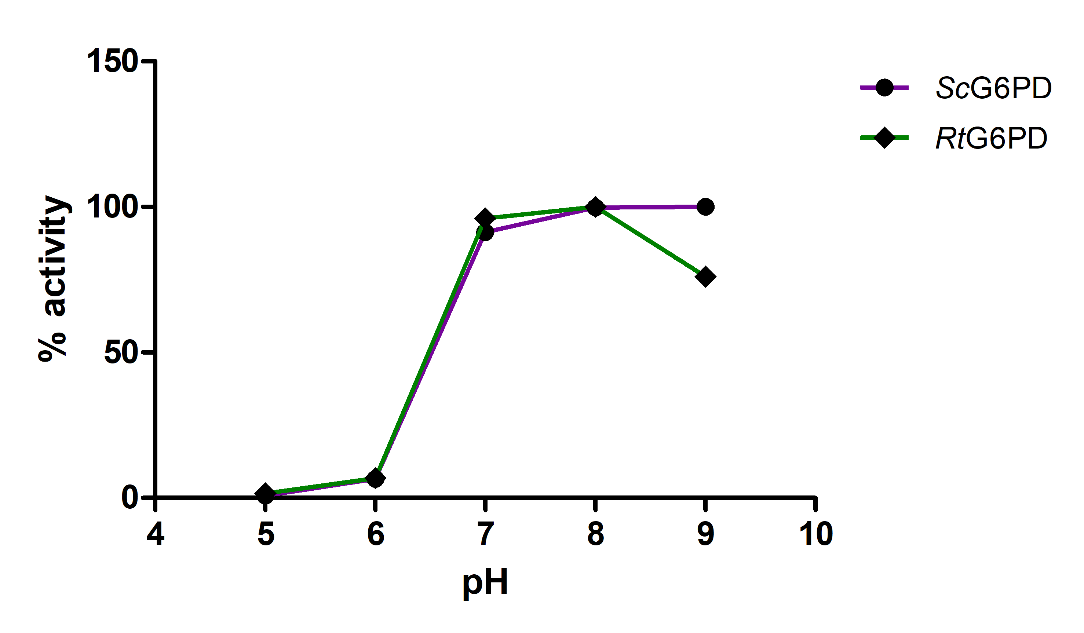


**Figure S2. Effect of pH on the activity of *Sc*G6PD and *Rt*G6PD.** The Relative % activity of *Sc*G6PD and *Rt*G6PD were compared at pH 5,6,7,8 and 9 in different buffers. pH 5 and 6 (phosphate buffer 100 mM), pH 7, 8 and 9 (Tris buffer 100 mM)


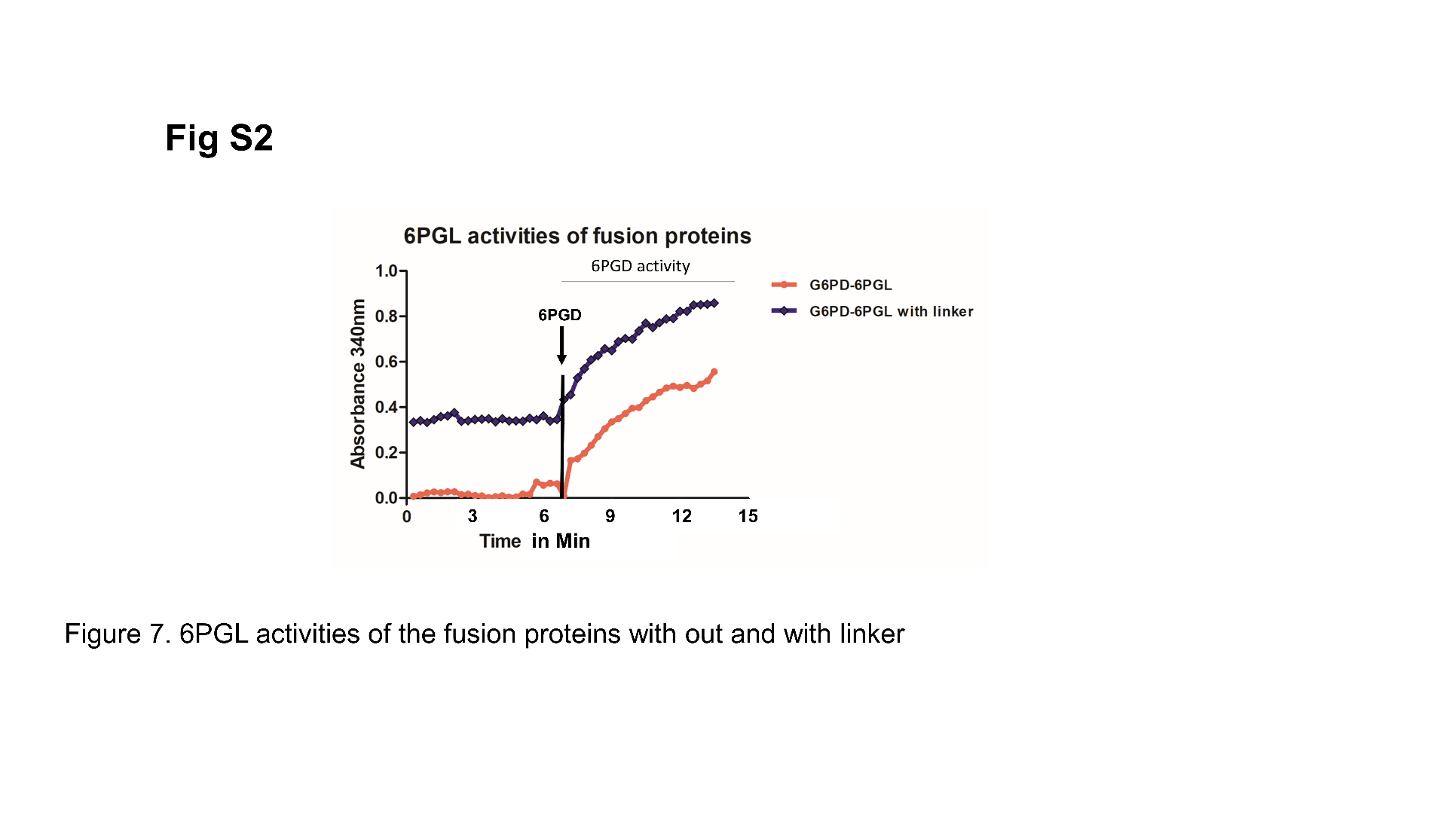


**Figure S3. 6PGL activities of the fusion proteins without and with a linker.** The fusion proteins were assayed with G6P and NADP. The G6PD domain would yield 6PGL substrate which would be acted upon by 6PGL domain, then the fusion enzyme was separated by a 30kDa amicon filter and the reaction mixture was incubated for 5 min, then 6PGD and NADP were added and increase in NADPH was monitored


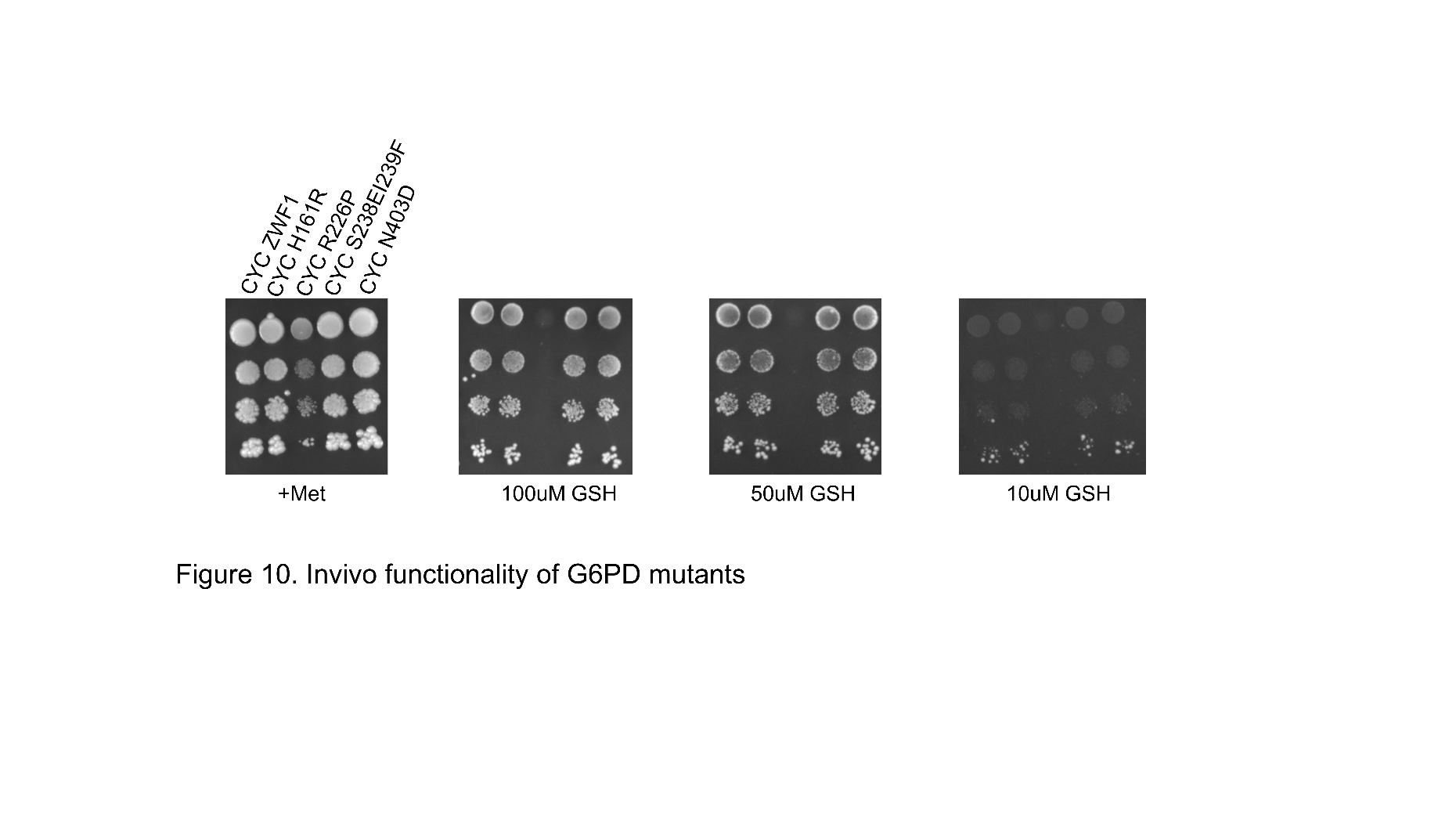


**Figure S4. *In vivo* functionality of G6PD mutants*.*** The different G6PD mutants constructed were expressed under weak CYC promoter, and growth was compared with the wild-type G6PD in *Δzwf1Δmet15* strain.


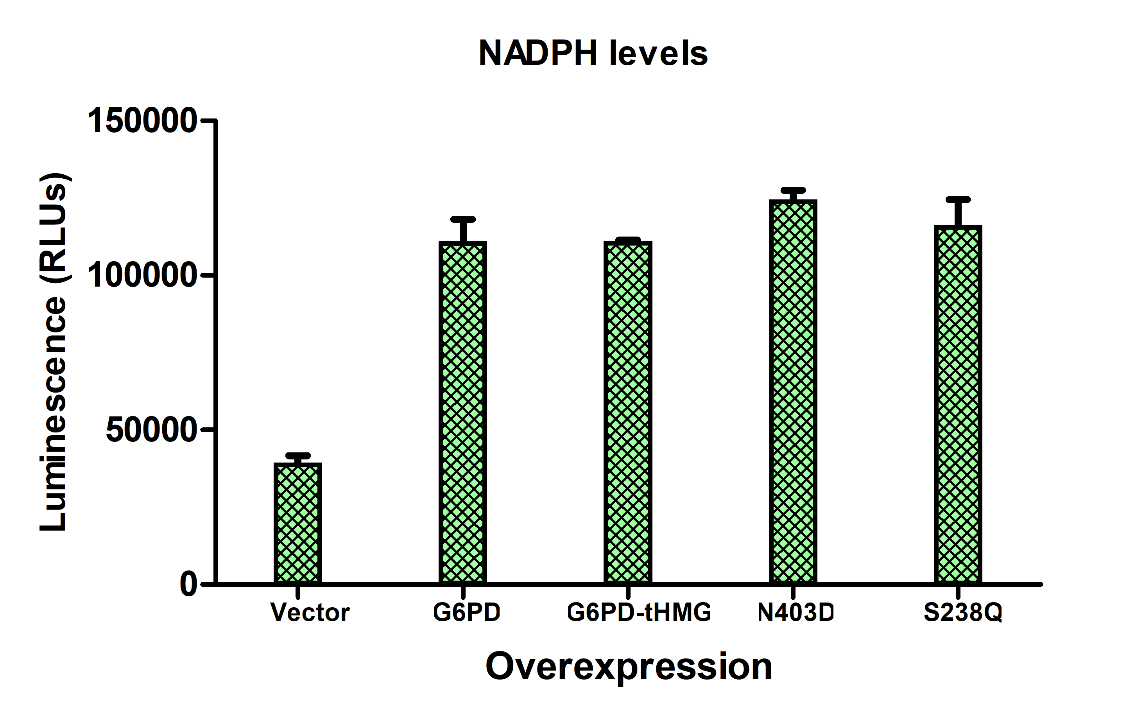


**Figure S5. Estimation of total NADPH and NADP pools *in vivo.*** NADPH and NADP pools were estimated using NADP/NADPH-GloTM assay kit. The graph shows a representative data set of two biological replicates. Error bars indicate SD.


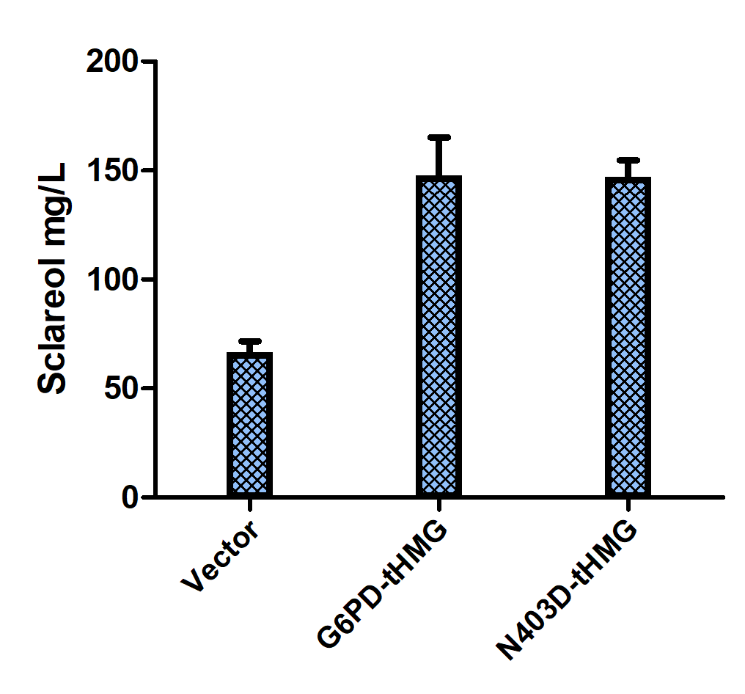


**Figure S6. Estimation of sclareol yields in strains overexpressing G6PD-tHMG and G6PD N403D mutant–tHMG fusion proteins.** Sclareol levels were estimated in the strains containing sclareol biosynthesis genes and overexpressed with G6PD-tHMG and G6PD N403D mutant–tHMG fusion proteins by GC-MS analysis. The graph shows a representative data set of three biological replicates. Error bars indicate S.D.


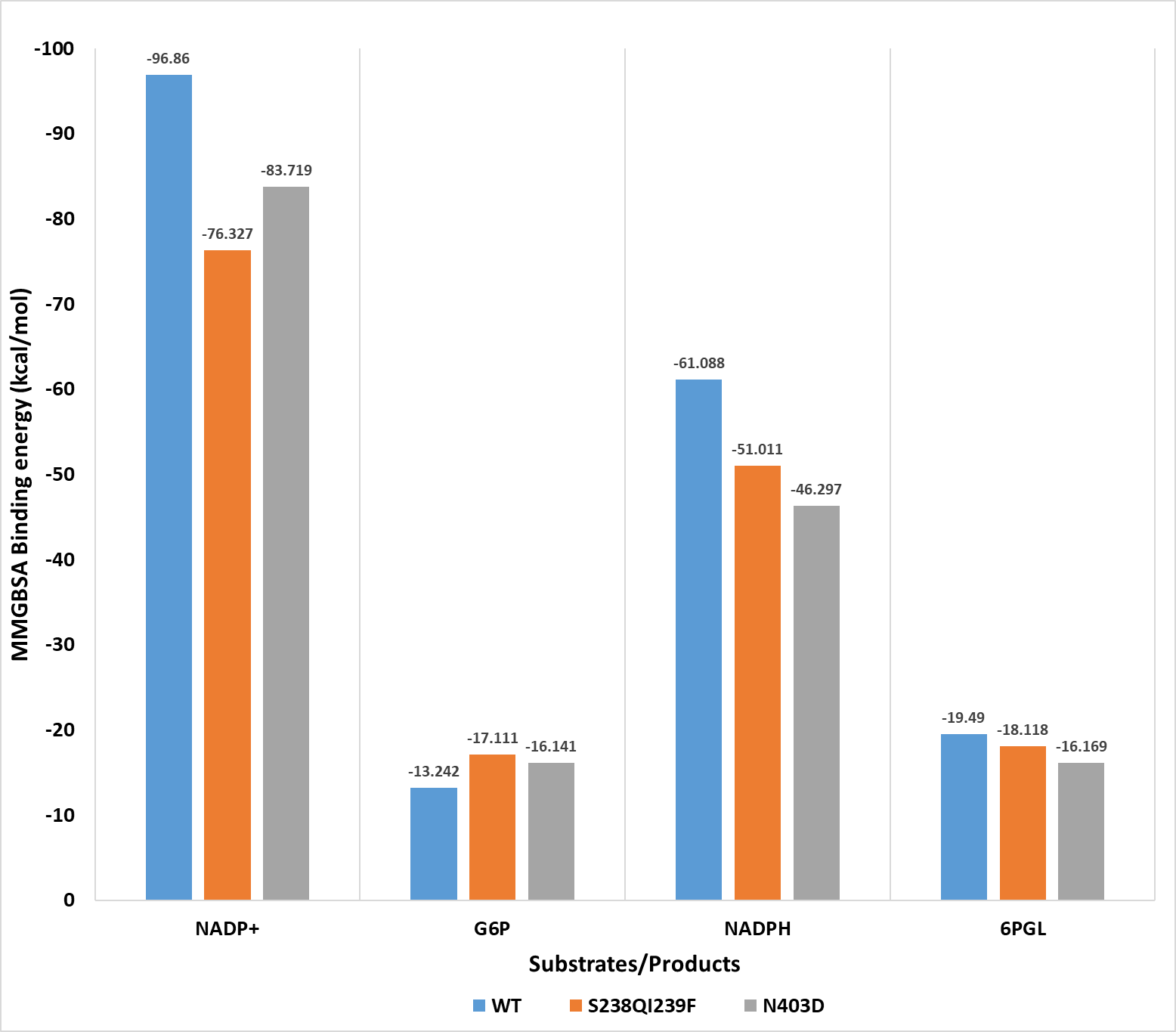


**Figure S7**. **MM/GBSA Binding energies of the substrate (G6P and NADP+) and product (NADPH and 6PGL) molecules with the wild type (WT) and mutant (S238QI239F and N403D) G6PD structures.**
